# Supplementary material for: Ultrasound trapping and navigation of microrobots in the mouse brain vasculature
Source: Nat Commun. 2023 Sep 21;14:5889. doi: 10.1038/s41467-023-41557-3 (PMC10514062; doi:10.1038/s41467-023-41557-3)
Supplement: Supplementary file 1 — Supplementary Information [file 41467_2023_41557_MOESM1_ESM.pdf]

Supplementary Information for

Ultrasound trapping and navigation of microrobots in the mouse brain vasculature

Alexia Del Campo Fonseca[1], Chaim Glück[2], Jeanne Droux[3], Yann Ferry[1], Carole Frei[1], Susanne Wegener[3], Bruno Weber[2], Mohamad El Amki[3#*], and Daniel Ahmed[1#*]

[1] Department of Mechanical and Process Engineering, Acoustic Robotics Systems Lab, ETH Zurich, 8092, Switzerland

[2] Institute of Pharmacology and Toxicology, University of Zurich, Winterthurerstrasse 190, 8057 Zürich, Switzerland

[3] Department of Neurology, University Hospital and University of Zurich, and Zurich Neuroscience Center, Zurich, 8091, Switzerland.

Daniel Ahmed
Email: dahmed@ethz.ch

This file includes:

Supporting text
Supplementary Figures 1 to 26
Supplementary References

Microbubble composition and fluorescence. During all the experiments we have used commercially available gas filled microbubbles (USpheres). These microbubbles are formed with a phospholipid outer layer that contains an inert gas on the inside, specifically, perfluoro propane (C3F8), see Supplementary Fig. 1.

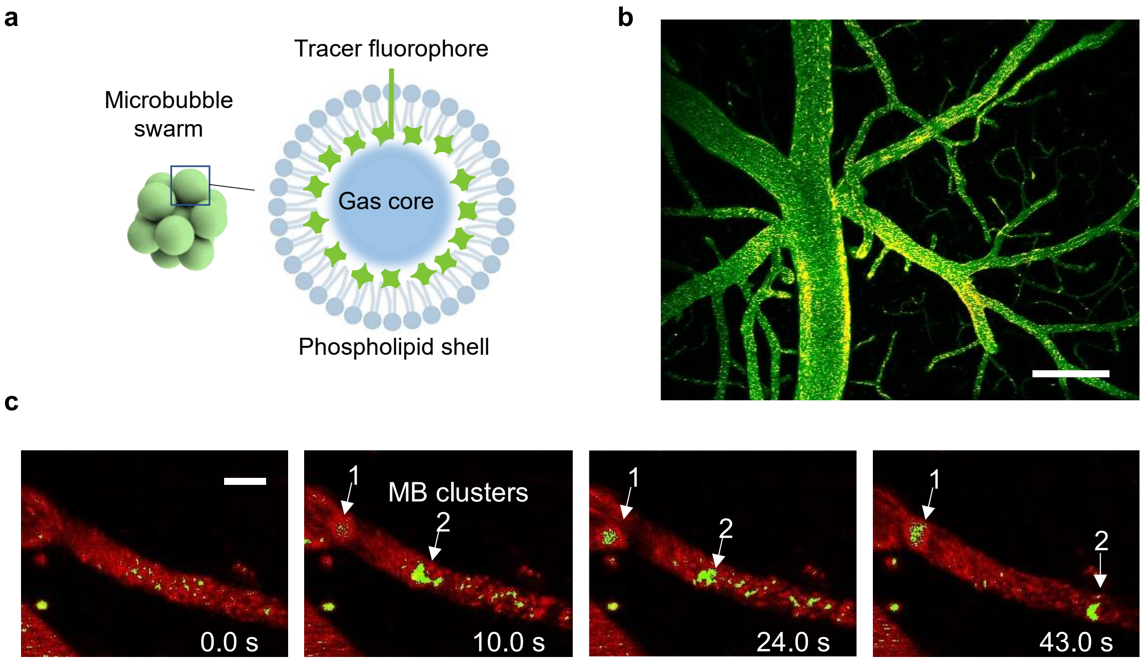

Supplementary Figure 1. **Microrobot and microbubble (MB) composition inside the pial vasculature of the brain. a.** Microrobots are composed by an aggregation of microbubbles. We used USpheres, which are microbubbles with a lipid coating and perfluoro butane gas in the inside. We manipulated and visualized microbubble swarms inside the pial vasculature of the brain. Phospholipid shell was created with BioRender.com. **b.** Two-photon (2P) microscope image of the pial vasculature of mouse brain. Bright green dots are the microbubbles flowing inside the vasculature. Scale bar is 100 µm. **c.** Images from the 2P microscope visualization of the pial vasculature of the brain when ultrasounds are activated. Microswarms have formed and they move along the vessel walls. The microswarms have been colored green; see Image processing in Methods section of the manuscript. This behavior was reproduced for at least 10 independent recordings. Scale bar, 30 µm.

Fluorescent microbubble dosage and the level of acoustic response

Note that two doses of 50 µl of microbubbles were injected from the tail vein of the mouse (concentration of ~2.5x1010 bubbles/ml) and acoustic actuation happens on the brain. It has been shown that the brain of a mouse constitutes 3.5 – 4% of the total blood volume[1], thus we can estimate that an order of 10^7 bubbles are circulating inside the mouse brain vasculature.

From our in vivo experiments, we computed the percentage of bubbles in circulation that displayed an acoustic response, which was manifested by their tendency to form swarms. Initially, we determined the number of swarms that had formed within the vessels. Then, we measured swarm diameter, and we calculated their volume assuming they had a 3D spherical shape. We approximated the quantity of bubbles forming each swarm by utilizing the diameter of a single bubble, as reported by USpheres, which was 1.1-1.4 µm. In addition, we possessed knowledge regarding the concentration of microbubbles present in the bloodstream, as well as the corresponding blood flow associated with each vessel. Consequently, we were able to calculate the proportion of bubbles that formed a swarm out of the total number of bubbles that flowed through each vessel. Importantly these measurements were taken from experiments in pial vessels of the brain.

Our analysis on the percentage of bubbles exhibiting an acoustic response was found to vary depending on the blood flow of the vessel. These results are discussed later and displayed in Supplementary Fig. 20. We also conducted a correlation analysis between vessel types, vessel diameters and the percentage of swarm formation. These results are shown in Supplementary Fig. 21. During our experiments we could trap into swarms a maximum of 20% from the circulating bubbles in pial vessel, mostly in capillaries and venules; while bigger vessels like arteries could show values down to 0.5% of bubbles trapped into swarms. In average, we obtained 3% of clustering from the circulating microbubbles in each vessel, during the time of recording. This means an order of 10^6 bubbles will be under the influence of the acoustic signal during the experimental time, if we could excite the whole brain acoustically. It's important to note that 2P microscopy does not allow for real time imaging at deeper regions of the brain. Thus, current

measurements can only be considered for superficial regions. We couldn't validate these predictions for deeper regions.

Positioning of piezoelectric transducer on top of a mouse skull. We activated the transducer at the excitation frequency of 490 kHz. Although in the present, there is a broad use of focused ultrasound techniques that can place the transducer separated from the skull [2–5]; here we are investigating the acoustic response of microrobots in the absence of a focusing force; thus the transducers had to be coupled directly to the bone.

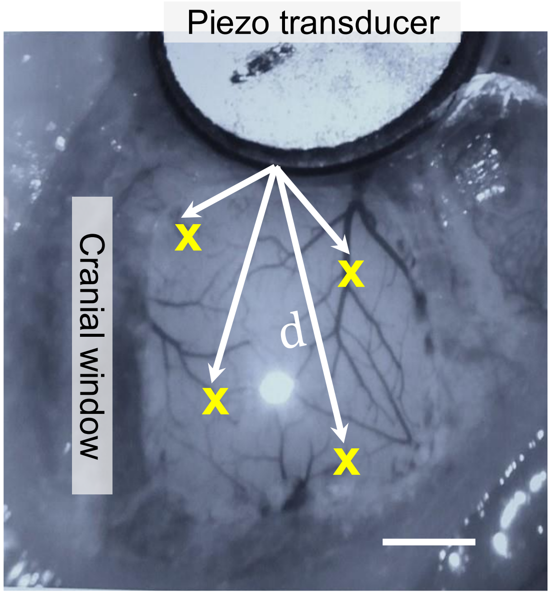

Supplementary Figure 2. **Variability introduced from distance and orientation between transducer and blood vessel.** Experiments in vivo were performed every time at similar tissue depths, however, we screened the whole cranial window (3x3 mm). In this image we see the cranial window from above and a piezoelectric transducer coupled on the top side. Thus, in each experiment we have a different distance '*d*' (distance to the transducer) and different angle '*α*' (angle between acoustic wave propagation and blood vessel orientation). This variability is translated into different microswarm formation rates and different swarm speeds inside the blood vessels. The experiments we performed didn't track the position of the field of view; thus, we work with this intrinsic variability. Scale bar is 1 mm.

Mass loading during transducer-skull coupling

When a mass is added to a piezo transducer, it affects its resonance properties by altering the resonant frequency and the mechanical impedance of the transducer[6].

The resonant frequency of a piezo transducer is determined by its physical dimensions and the elastic properties of the materials used in its construction. When a mass is added to the transducer, it increases the effective mass of the system and changes the resonant frequency. Specifically, the resonant frequency of the transducer decreases as the added mass increases[6].

The mechanical impedance of a piezo transducer is a measure of its resistance to deformation under an applied force. It is directly related to the resonant frequency and the effective mass of the transducer. When a mass is added to the transducer, it increases the effective mass and reduces the mechanical impedance of the system. As a result, the transducer may become less efficient in converting electrical energy into acoustic energy[6].

Gluing a piezo transducer to a surface introduces mass loading to the system and affects its resonance properties; this is exactly what we see in Supplementary Fig. 3. During our experiments, we glued a piezoelectric transducer to a mouse skull, thus increasing the total vibrating mass. The amount of change in resonant frequency will depend on the amount of mass added and the stiffness of the surface[7].

Measurement of the intracranial pressure field

The skull of mice has between 1-2 mm depth and it's followed by protective meningeal layers and cerebrospinal fluid before reaching the brain tissue [8,9]. The main role of meningeal layers is to protect our brain from physical trauma, thus they act as shock absorbers[9]. Although these layers are very thin, they will still contribute to the attenuation of the acoustic wave. We need to consider that the acoustic pressures we measured below the skull, do not include the contribution from the meningeal layers. Even so, we studied the feasibility for the remaining acoustic pressure to activate microrobots.

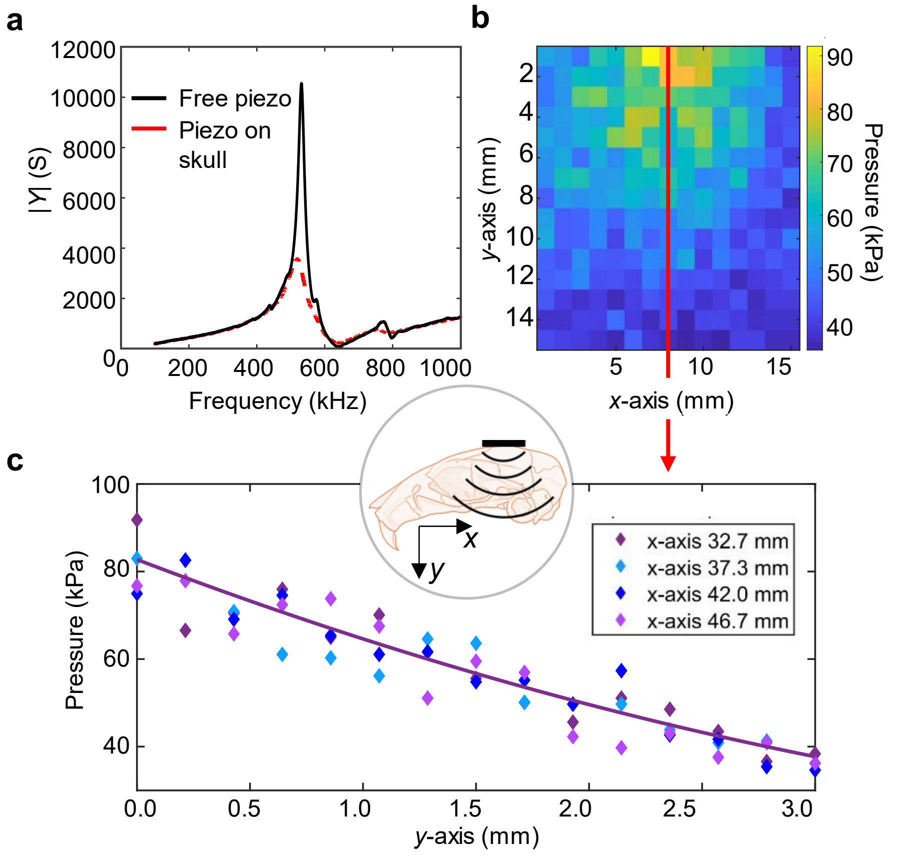

Supplementary Figure 3. **Acoustic pressure measurements within an *ex vivo* mouse skull. a.** Impedance analysis of the piezoelectric transducer (490 kHz resonance frequency, as detailed by the provider) with and without skull coupling. An impedance analyzer has been used to measure the impedance of the transducer given an excitation frequency. **b.** 2D map of the acoustic pressure below the skull upon transducer activation at 490 kHz and 45 V_{PP}. For more details on the

measurement procedure, see Methods. **c.** 1D acoustic pressure along the *y* axis of the skull at different *x* values. Each point is a single measurement taken by the hydrophone at a point in space. A general attenuation decay is observed, reflected by the best-fit curve (purple line). R^2 =0.8802 was used for fitting assessment. Inset shows a schematic of the mouse skull, definition of *x* and *y* axes, and transducer position during measurements. Mouse skull was created with BioRender.com.

Simulations of acoustic pressure below a mouse skull. A numerical model of a rodent skull and brain was designed to evaluate the spatial distribution of the acoustic field inside the cortex of the animal. These data would provide an overview for the upcoming in vivo study.

To have a better overview of the acoustic field map below a mouse skull, it has been decided to pursue the study with a 3D model, see Supplementary Fig. 4. The skull geometry is taken from a 3D scan of a juvenile female mouse made available through ONE Core facility. We added to the geometry 3 5*5*0.4 mm piezo elements and a fluid-filled cavity representing the brain (COMSOL 6.0). An appropriate mesh for the finite element analysis was built based on the physics used in the finite element analysis software.

The numerical model uses 3 physics modules (acoustic, mechanic, and electrostatic) for 3 different domains (skull, brain, and piezo elements). Piezo elements were modelized as PZT-5A material and simulated by coupling mechanic and electrostatic physics module. They were numerically excited at a frequency of 490 kHz and an amplitude of 25 V_{PP}. The propagation of the acoustic waves due to piezo transducer motion has been computed with the mechanic module. The displacements generated are below the micrometer range and the strains are lower than 10^{-3}, thus a linear elastic material model complemented with damping has been chosen to model the skull bone. Lastly, the acoustic field in the skull cavity has been computed with the acoustic module and appropriate damping. The skin has not been taken into account here as in our in vivo experiment the mouse cranial epidermis was removed to build a cranial window.

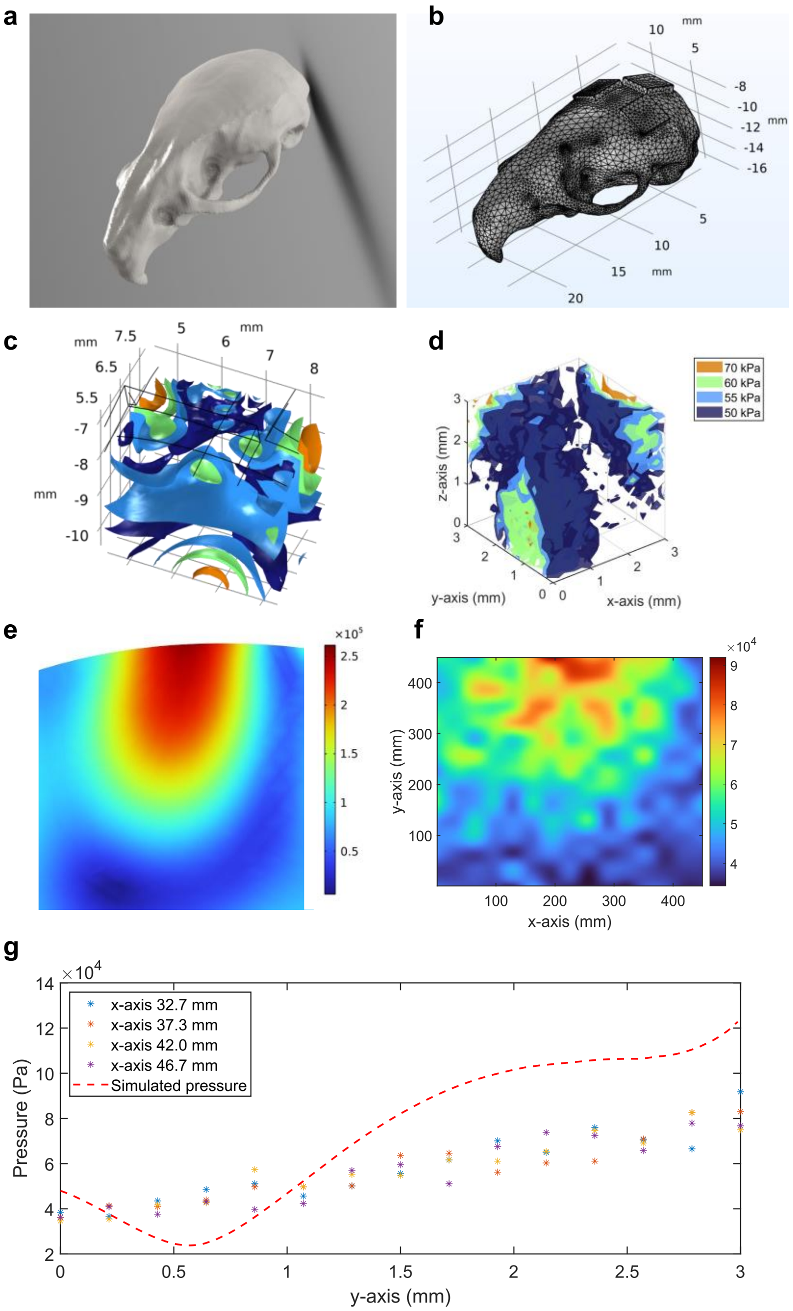

Supplementary Figure 4**. Acoustic pressure simulation results and its comparison with experimental measurements. a.** Skull scan .stl file from ONE Core. **b.** Skull import in COMSOL, with three piezo elements added on top of the skull and meshing of the whole geometry for finite element analysis. **c.** Intracranial pressure field computed by the numerical simulation below three piezo transducers. **d.** Interpolated intracranial pressure field experimentally measured below three transducers. **e.** *X-Y* plane of the simulated pressure field at an antinode. **f.** *X-Z* plane of the

measured pressure field at an antinode. **g.** Plot comparing the evolution of the simulated versus measured pressure field along the y-axis and centered at the maximum pressure along the x-axis.

Our numerical model was compared with a 3D scan (3*3*3 mm) of the intracranial pressure field in an Ex Vivo rodent, see Supplementary Fig. 4. As in the simulation, the three piezo transducers were excited with 10 cycles pulse at a frequency of 490 kHz and an amplitude of 25 V_{PP}. A particular pattern of 3 pressure antinodes has been observed analogous to the results output by the simulation. The order of magnitude is quite similar. Lastly, if we compare the measured and simulated pressure distribution along a *y*-axis line, we observe that absolute pressure amplitude ranges from 70 kPa to 100 kPa at the interface between the skull and brain cavity, Supplementary Fig. 4.

Acoustic manipulation of microbubbles through thick bones

We have tested the validity of these piezo transducers for the manipulation of bubbles through thick skulls. For this experiment we obtained a piece of bone from a pig knee (acquired from a local butcher store) with 5.33 mm of thickness. We glued a transducer to one side of the bone, and we glued the other side to PDMS. We activated the transducer at 490 kHz and increasing voltage from 2 to 10 $V_{PP,}$ Here, we demonstrated that even at low voltages, the acoustic signal passes through the bone to the microchannel, leading to microswarm formation and navigation. Microbubble velocity was observed to increase with the voltage applied, see Supplementary Fig. 5.

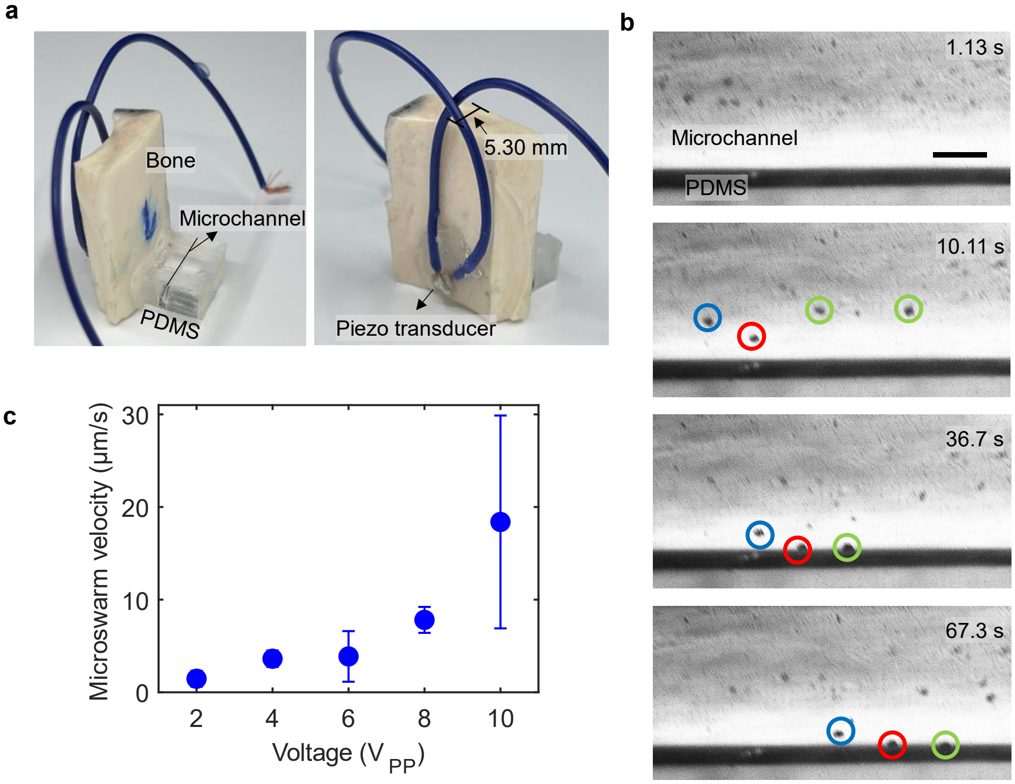

Supplementary Figure 5. **Actuation of microbubbles through a thick bone. a.** Experimental set-up. A bone of 5.30 mm thickness was placed in between the piezo transducer and the PDMS with the microchannel. See scale bar on the bone, 5.3 mm. **b.** Image sequence showing microbubbles forming swarms and moving at 490 kHz and 10 V_{PP} of actuation signal. **c.** Voltage characterization. Swarm velocities inside this set-up were analyzed at increasing voltages. The error bar represents the standard deviation from the mean value and each point represents the average of 5 measurements taken for each voltage value. Scale bar is 100 µm.

Acoustic behavior of microrobots versus distance to the transducer

The pressure associated to acoustic waves naturally decays with distance in air, as explained by the inverse square law[10]. Here we derived the relation between acoustic radiation force, microbubble velocity, acoustic pressure and distance to the acoustic source, to clarify how microbubble navigation velocities can be affected by the distance of their vessel to the acoustic source. However, note that acoustic waves are also dampened by the material where they are traveling, and by the presence of obstacles and inhomogeneities within their trajectory[11]. The results obtained from experiments in PDMS demonstrate the tendency of velocity to decay with distance, but the exact decay rate will change when implementing it in vivo.

If we assume that the acoustic pressure follows an inverse proportionality with distance from the source, then we can write:

$P = P_0/r^2$ (1)

where *p0* is the pressure at a reference distance and *r* is the distance to the acoustic source.

The acoustic radiation force, **F**, acting on a microparticle in an acoustic field is given by:

$F = k\nabla P$ (2)

where *k* is the particle's acoustic contrast factor and *∇p* is the gradient of the acoustic pressure.

To calculate the gradient of the acoustic pressure, we can take the partial derivatives with respect to the Cartesian coordinates *x, y*, and *z*:

$\frac{\partial P}{\partial x} = -P_0 x/r^2$, $\frac{\partial P}{\partial y} = -P_0 y/r^2$, $\frac{\partial P}{\partial z} = -P_0 z/r^2$ (3)

where *p0x, p0y*, and *p0z* are the components of the acoustic pressure vector at the reference distance. The magnitude of the gradient of the acoustic pressure is then:

$|\nabla P| = sqrt\left(\left(\frac{\partial \mathrm{p}}{\partial \mathrm{x}}\right)^2 + \left(\frac{\partial \mathrm{p}}{\partial \mathrm{y}}\right)^2 + \left(\frac{\partial \mathrm{p}}{\partial \mathrm{z}}\right)^2\right) = P0/r^2$ (4)

Substituting this expression into the relationship between the acoustic radiation force and the gradient of the acoustic pressure, we get: $F \propto P_0/r^2$ (5)

which simplifies to: $F \propto 1/r^2$ (6)

This shows that the acoustic radiation force decreases with the square of the distance from the source. The velocity, **v**, of a microparticle subjected to a constant force, **F**, can be related to the distance, *r*, from the acoustic source using Newton's second law:

$F = ma = m(\frac{dv}{dt})$ (7), where *m* is the mass of the microparticle and *a* = *dv/dt* is its acceleration. Assuming that the force **F** is constant, we can integrate the above equation to obtain: $v = \left(\frac{F}{m}\right)t + v_0$ (8), where *v0* is the initial velocity of the particle. Substituting the relationship between force and distance, we get: $v = \left(\frac{P_0}{mr^2}\right)t + v_0$ (9)

To this decay, we need to add material and inhomogeneities effects. During our experiments, microbubble clusters of size 23.7 ± 4.3 µm decreased their velocity with distance. See Supplementary Fig. 6.

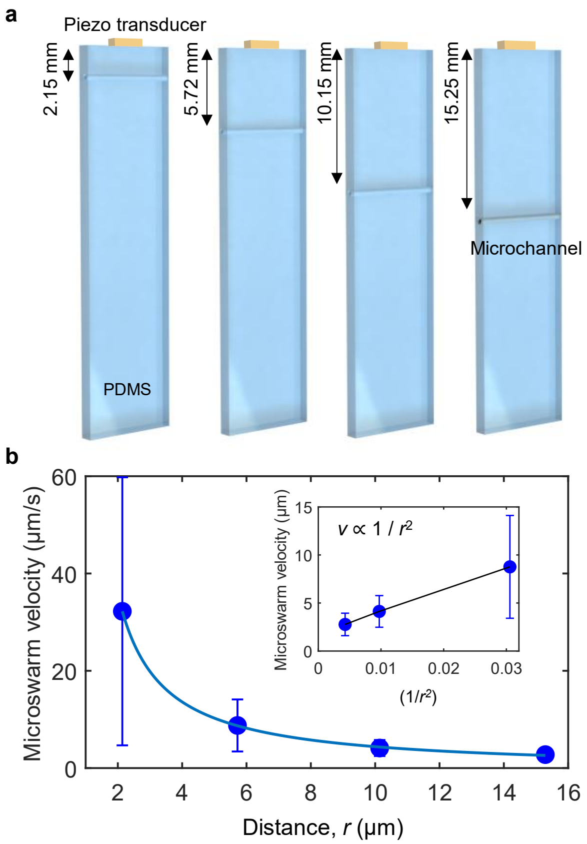

Supplementary Figure 6. **Microbubble navigation velocity at microchannels with different distances from the piezo transducer. a.** Schematic of the experimental set-up. Four different set-ups were used with channels at four different distances, detailed in the image. The piezoelectric

transducer is placed on the top short end of the PDMS device. **b.** Microbubble velocity, *v*, during swarm navigation versus distance to the transducer, *r*. Swarms measured had size of 23.7 ± 4.3 µm to avoid velocity fluctuations due to size. Every point represents the average of five measurements and the error bars represent the standard deviation from the mean value. In light we have plotted a fitting line (blue) that follows a quadratic inverse proportional fit. The logarithmic analysis is shown as a subplot, demonstrating the quadratic fit, R^2=0.9996.

Microbubble navigation in vertical channels

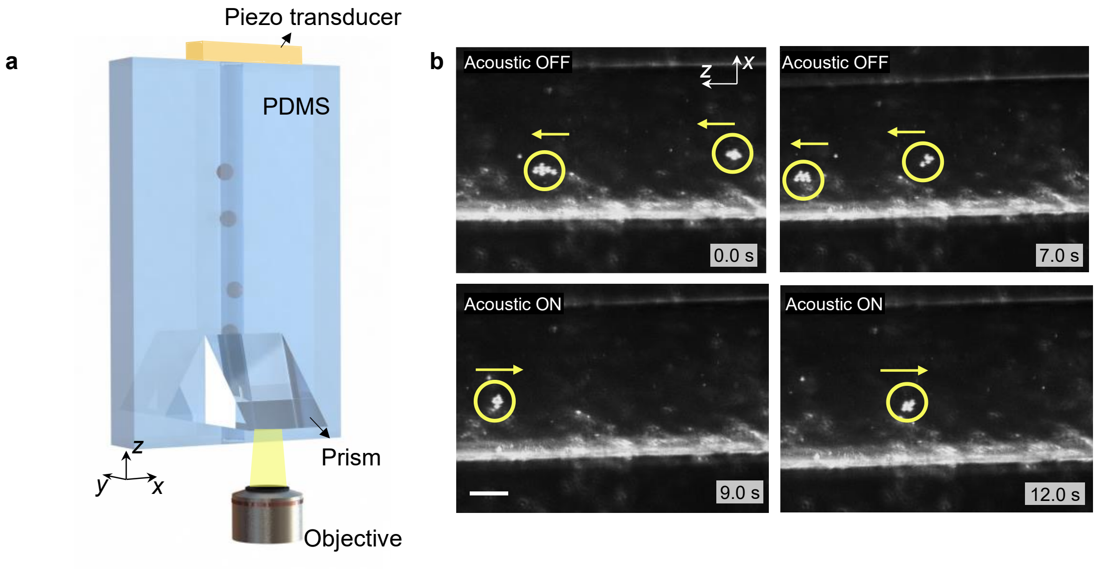

Supplementary Figure 7. **Microbubble navigation in vertical channels. a.** Schematic of the experimental set-up, a glass right angle prism was coupled to the face of the PDMS microfluidic device, and it was oriented towards the light of the microscope. On the camera that was mounted to the inverted microscope, we observed a tilted image, where the top side of the PDMS is located at the left of the visualization window (see images in b). **b.** Microscope time sequence images of microbubbles (yellow circles) inside the vertical channel under acoustic actuation of 490 kHz and $10V_{PP}$. During Acoustic OFF phase, microbubbles move naturally to the left (to the top of the channel), guided by buoyancy effects. Upon acoustic activation, microbubbles form a swarm, they attach to the side wall and then move to the right (to the bottom of the channel). For these

experiments the transducer was placed at the top (so acoustic wave propagated downwards). This behavior was reproduced for 5 independent experiments. Scale bar is 100 μm.

Microbubble self-assembly and navigation using uniquely one transducer.

In previous studies, the acoustic navigation of microbubble swarms in vitro was shown as a two-step actuation that required two different piezoelectric transducers.[12] A first transducer located parallel to the artificial vessel, was used to activate microbubble swarm assembly and its movement towards the vessel wall. And a second transducer perpendicular to the vessel was employed to ensure propulsion along its length. Here, we use the same acoustic navigation principle, however we prove that the system can be simplified to a single transducer that achieves all, assembly, adherence to the wall and propulsion. This simplification is necessary for the implementation of this system in vivo, otherwise for each vessel's relative position, we would need two specific transducers to ensure manipulation.

The aggregation of microbubbles into swarms results from microbubble oscillations when they are inside an acoustic field. The oscillations scatter the sound in all directions, creating a pressure gradient that attracts microbubbles to each other and thus microbubble assembly takes place. Importantly, this attraction occurs irrespective of the acoustic field's source, facilitating microbubble assembly regardless of the field's location. Similarly, the secondary Bjerknes forces that drive microbubble attraction to a wall are also independent of the acoustic field's source. Although the presence of a travelling acoustic wave directed towards the vessel wall can aid microbubbles in moving directly towards the wall, this wave is not required for wall attraction. When microbubbles are in close proximity to the wall, they are driven towards it by the wall's attractive force.

Using the same experimental setup as the previous study, we have demonstrated our findings once again, but this time using a single perpendicular transducer (as shown in Supplementary Fig. 8). The microfluidic device comprises a channel with a square cross-section of 400 x 30 μm, made of PDMS material, and the piezoelectric transducer is 242 kHz. Here we observe that microbubbles initially self-assemble and move in the direction of wave propagation. The movement of

microbubbles is not perfectly straight, and as soon as they get close to the wall, they attach to it, and they continue their navigation along the channel wall.

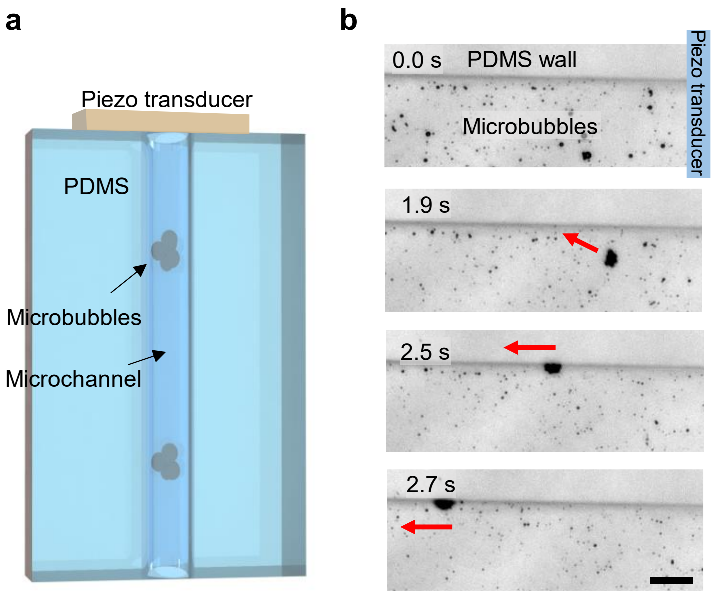

Supplementary Figure 8. **Formation and navigation of microswarms along a microchannel wall with a single piezoelectric transducer. a.** Schematic of the experimental set-up. A single transducer attached to the lateral wall of the PDMS was used for this proof of concept. **b.** Image sequence of microbubble behavior upon acoustic excitation at 242kHz and 3VPP. As seen in the various time frames, microbubbles assemble into a swarm, and they move in the direction of wave propagation. The swarms that come close to the PDMS wall feel the attraction from the secondary Bjerknes forces, and they adhere to the wall. Once at the wall, swarms keep moving in the direction of wave propagation. This behavior was reproduced for 5 independent experiments. Scale bar is 100 µm.

Further experiments in 3D microfluidic set-ups demonstrated that by activating each time one single transducer, we could achieve formation and navigation of swarms. Note that in the current system, there is more than one transducer, but each transducer is activated individually each time, with the final goal of steering the microrobot through branches or irregular pathways, see Supplementary Fig. 9. If our goal was only swarm formation and navigation in a straight line, the activation of one transducer would be enough.

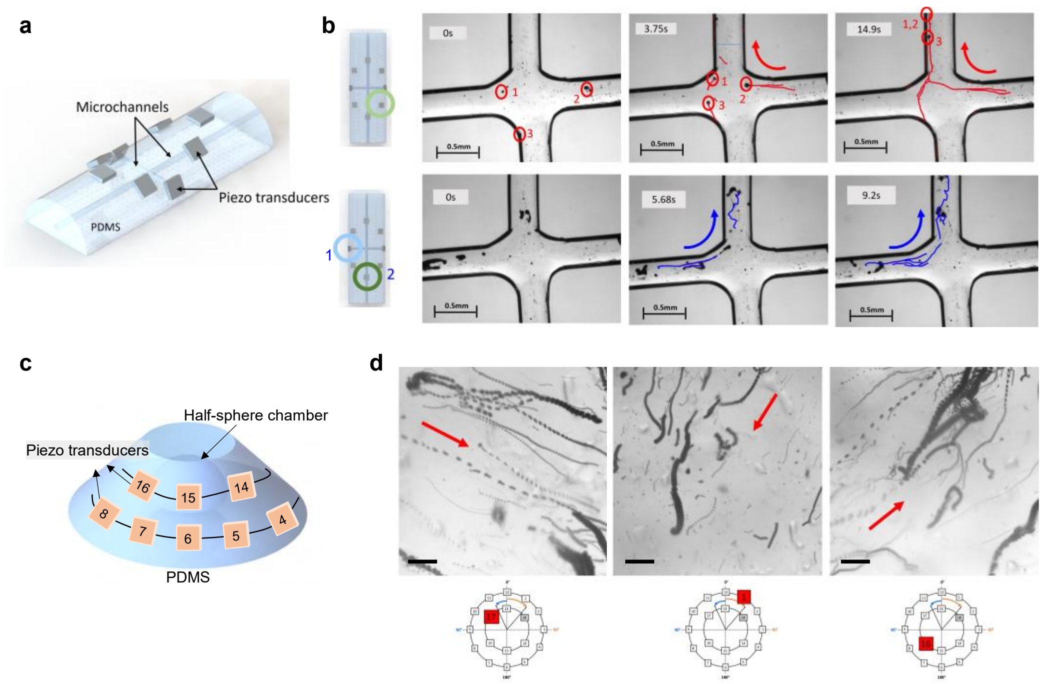

Supplementary Figure 9. **Formation and navigation of microswarms along defined trajectories with a combination of single piezoelectric transducers**. **a.** Schematic of the experimental set-up used for the experiment. 8 transducers were attached to a PDMS device. The microfluidic device contained a cross design mimicking an intersection between two vessels. **b.** Image sequence of microbubble behavior upon acoustic excitation at 242kHz and 3VPP of transducers. Transducers were activated in a sequential way, as marked by labels 1 and 2 in the left image. As seen in the various time frames, microbubbles assemble into a swarm, and they move in the direction of wave propagation. The swarms can move into the desired branches upon activation of the correct transducers. Similar behaviors were achieved in at least 5 independent experiments. Scale bar is 500 µm. **c.** Design A. Schematic of the cone frustum PDMS experimental set-up used for experiments. 18 transducers were attached to the PDMS device distributed in two rows. Inside the device we fabricated a half-sphere hollow chamber where we later injected a microbubble containing solution. **d.** Each image consists of a stack of images during acoustic activation. In black we see the microbubbles and in a red arrow we show the trajectory of microbubbles navigation when one transducer is activated. Below each image we have marked in red the activated transducer. Each transducer was activated 3 times getting reproducible results. Scale bar is 100 µm.

Microbubble navigation in curved channels

To further characterize the capabilities of navigation inside various trajectories, we investigated the manipulation of bubbles through curved channels, specifically a channel with an S-shape. We fabricated a PDMS device, in which a wire with an S-shape was embedded. After polymerization, the wire was pulled out and a channel with circular cross-section and 400 μm diameter was engraved in the device. We placed two piezoelectric transducers, as shown in Supplementary Fig. 10, and we activated them each time at $20V_{PP}$ and 490 kHz. Each transducer faced opposite directions, up or down, and we divided the S-shape channel in two curves, curve 1 was convex and curve 2 was concave. It is important to note that in order to achieve curved trajectories, it is necessary to move in two opposite directions along the trajectory. For instance, when navigating a convex curve, the microbubbles need to move upward until reaching the apex of the curve, and then downward on the other side. As a result, at least a combination of two transducers is required for effective microbubble navigation in curved trajectories. Upon initial observation, we noticed that the position of the curves and the piezo transducer in our setup allowed for only right-downward movement with Transducer A in the first curve. The acoustic wave from transducer B didn’t reach this area with enough intensity, and transducer A was slightly placed to the right, so left side didn’t show an organized movement pattern. Conversely, in curve 2, the positioning of the transducers was optimal for bubble manipulation. Transducer A was situated to the left of the curve, while Transducer B was positioned to the right. With Transducer A, bubbles were directed downward until they reached the middle of the curve. Then, Transducer A was deactivated, and Transducer B was activated, causing the bubbles to move upward to the other side of the curve. In conclusion, successful navigation through curved channels was achieved, which is particularly relevant as similar geometries are present in in vivo conditions.

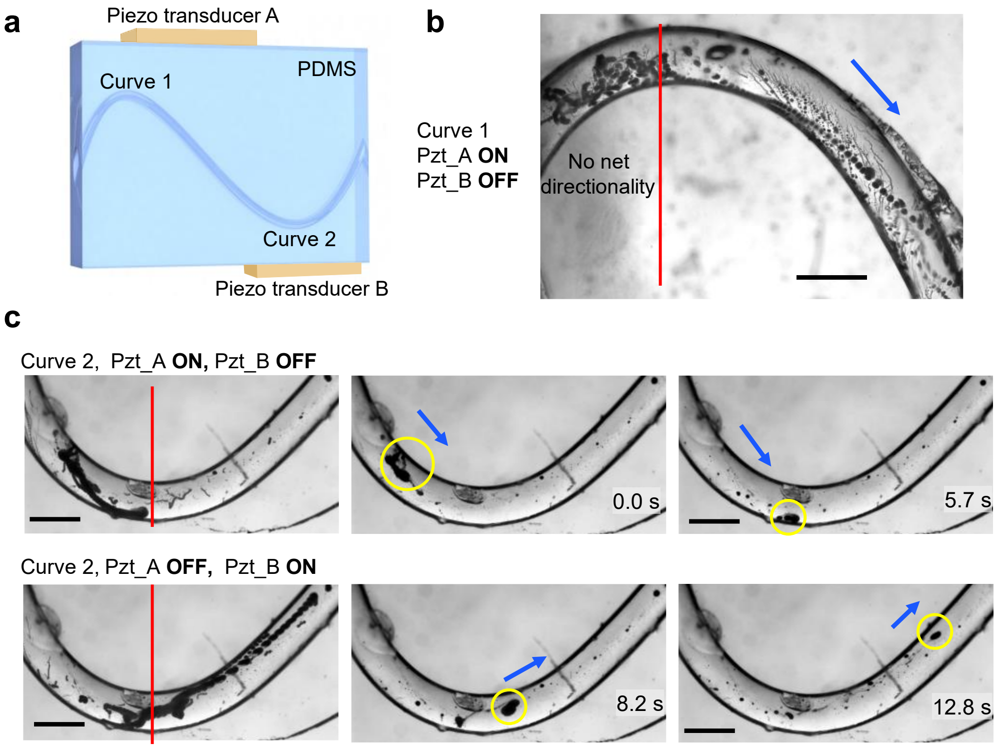

Supplementary Figure 10. **Microrobot navigation in curved vessels. a.** Experimental set-up. PDMS device with two transducers glued. The set-up was placed on an inverted microscope to facilitate visualization, ensuring that the vessel remains in the same plane and is unaffected by buoyancy when moving through the S shape. **b.** Movement of microbubbles along Curve 1, upon activation of Piezo transducer A at 20 V_{PP} and 490 kHz. The image shown is a stack of 200 frames taken with the microscope for 23 seconds. Microbubble trajectory is shown with a blue arrow. Scale bar is 400 µm. **c.** Movement of microbubbles along Curve 2, First activation of Piezo transducer A, at 20 V_{PP} and 490 kHz, moves microbubbles down-right. Then Transducer A is deactivated, and Transducer B is activated, so bubbles move top right. The images at the left are stack images during microbubble navigation. On the right we show the first and last frame of the navigation process, thus the first and last position of the microbubbles. Microbubbles are marked by a yellow circle and microbubble trajectory is shown with a blue arrow. This behavior was reproduced for 5 independent experiments. Scale bar, 400 µm.

Manipulation of microrobots for long distances

Our results in microfluidic vessels have shown that the microrobots can navigate continuously along paths up to 10.05 mm, as long as the acoustic wave hits them from an optimal direction with sufficient intensity, see Supplementary Fig. 11. However, in vivo experiments have presented challenges due to the complexity of the brain and the interactions of the acoustic wave with multiple tissues. We have observed that the coupling between the transducer and the mouse skull, as well as scattering of the wave at every interface, can compromise the intensity of the acoustic wave.

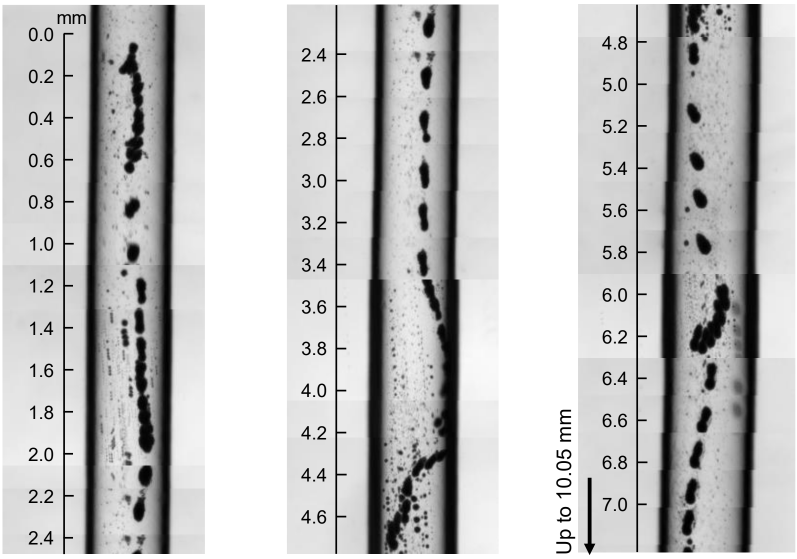

Supplementary Figure 11. **Continuous driving of acoustics microrobots.** A piezo transducer placed at the top of the image (perpendicular to the channel) was activated at 490 kHz and 10 V_{PP}. We tracked the microrobot down a channel that was 400 μm in cross section diameter and 15 mm long. On the left a scale in mm is shown. This behavior was reproduced for 5 independent experiments.

The simultaneous control of multiple swarms formed within the brain region.

This study uses a single transducer for the generation of the acoustic signal. The acoustic transducer generates a traveling acoustic wave that penetrates through the tissue and spreads along a defined region within the brain. In our study, we concentrated on a set of particular target vessels to which we directed the navigation of the microrobots. However, it is important to note that microswarms are also being generated concurrently in other vessels located within the acoustic region, and their characteristics are determined by their respective distance and orientation relative to the acoustic transducer.

To address this phenomenon, a comprehensive understanding of the precise location of all the vessels impacted by the acoustic wave is crucial. In order to achieve this, we utilized a 2P microscope to map the pial vasculature of the mouse, which represents the network of blood vessels located within the superficial region of acoustic actuation. We have processed the image to reduce noise and we have created a skeleton structure of the imaged vasculature; such that each single vessel is represented by a single line that follows the vessel trajectory in space, see Supplementary Fig. 12. Utilizing this skeletal structure, we extracted the orientation of each vessel, which refers to the angle between the vessel and the horizontal axis, as well as the distance between the vessel and the transducer.

We previously demonstrated that the angle and distance of the transducer is a key determinant of the direction in which microbubbles move, see Fig. 3. Therefore, by possessing advanced knowledge of the relative position of each vessel with respect to the transducer (i.e., its angle and distance), we are able to anticipate the behaviour of microswarms in each vessel, including their direction of movement and velocity, see Supplementary Fig. 12-18. For this goal, we made the assumption that the piezo transducers were arranged in a circular formation and directed towards the centre. Using this assumption, we determined the relative positioning of each vessel with respect to each transducer in our system. It should be noted that the calculations remain consistent regardless of the number of transducers used. As discussed throughout the manuscript, the optimal placement of the transducer for swarm navigation is perpendicular to the channel, where the line connecting the center of the circumference to the transducer is parallel to the vessel. At this

configuration, the swarm velocity is at its maximum, and as the angle deviates from this position, the velocity gradually decreases. Consequently, for each transducer activation, we can identify the vessels that will be most significantly impacted, see Supplementary Fig. 13.

Once we have stored data on the spatial relationship between the activated transducer and the blood vessels, we used this data to anticipate the path that microrobots would traverse within each vessel following activation of a designated transducer. Upon activating each transducer, microrobots tend to move in the direction of wave propagation. This behavior predominantly occurs in vessels that are appropriately aligned with the wave propagation direction. To visually convey this information, we generated images of the reconstructed vasculature network, where different colors indicate different vectorial directions of the microrobot trajectory, see Supplementary Fig. 14. For example, if a transducer is located at 180° (see angle reference in Supplementary Fig. 14a), the microbubbles will move to the (1,0) vectorial direction. Thus, in this case, blood vessels where microbubbles are present, will be colored in cyan blue. Within these images, the intensity of the color serves as an indicator of the effective micro vessel orientation. Brighter vessels correspond to those in which microrobots exhibit increased movement and higher velocity. For our example, those vessels located horizontally in the image will show bright blue colors, while vertical vessels will show the darker tones of blue (see Supplementary Fig. 14c)

Within the vessels that exhibit a more favorable orientation in relation to the transducer, there will also be relative changes in velocity. These changes are attributed to the varying distances between the vessels and the transducer. Thus, we additionally depicted the velocity change that occurs between different blood vessels due to their relative distance to the activated transducer. As explained in Supplementary Fig. 6, microbubble velocity decreases as their distance from the transducer increases. We acknowledge that acoustic pressure diminishes with increasing distance from the transducer. Furthermore, we recognize that acoustic pressure directly correlates with the velocity of microbubbles, as depicted in Supplementary Fig. 6. Leveraging the knowledge of the distance between the transducer and each blood vessel, we generated a reconstructed image of the vasculature network where brighter colors indicate shorter distances to the transducer, and as

consequence higher microbubble velocities, see Supplementary Fig. 15. It is noteworthy to mention that our experimental measurements, as illustrated in Supplementary Fig. 3, include acoustic pressure readings beneath a mouse skull at various depths and distances; we also include this information in Supplementary Fig. 15. The maximum distance from the transducer within our field of view is half a millimeter. Notably, we observed a small decay in experimental acoustic pressure within this distance range. Ultimately, by combining the information from Supplementary Fig. 13, 14, 15; we can derive the vessels where microrobot formation will take place, their velocity and their movement direction.

On our study we have applied this approach to the vasculature maps of mice and reconstructed the skeleton structure of the vasculature map using a color map, where vessels appearing brighter indicate a higher degree of impact from the actuated piezo. The experiments in vivo proved that the identified brighter vessels showed microswarm formation events, while the darker vessels didn't, see Supplementary Fig. 16. In addition to the aforementioned validations, we have further confirmed that the formed microswarms exhibit movement in the direction of wave propagation. This validation is supported by the findings presented in Supplementary Fig. 17, which provide visual evidence of the directional movement of the microswarms aligned with the wave propagation. Furthermore, we have also validated the velocity and effectiveness of microswarm movement when the microswarms are positioned in close proximity to the transducer. Fig. 4 demonstrates the improved movement efficiency of the microswarms in this scenario.

At present, the predictions derived from vasculature imaging assist us in designing an optimized transducer configuration for the targeted vessels. We anticipate that in the future, an automated processing system capable of analyzing the entire vasculature network architecture, combined with machine learning techniques, will permit the simultaneous control of multiple microswarms via dynamic modulation of the acoustic signal.

However, there are still challenges to face. Despite significant advances in imaging techniques, limitations still exist when it comes to examining deeper regions of the brain. Real-time imaging of microscale objects inside deeper tissue areas using minimally invasive approaches is incredibly

challenging. As a result, we were unable to analyze swarm formation and navigation in deeper regions during our studies, and although the predictions we made can be extended to them, we were unable to verify their validity.

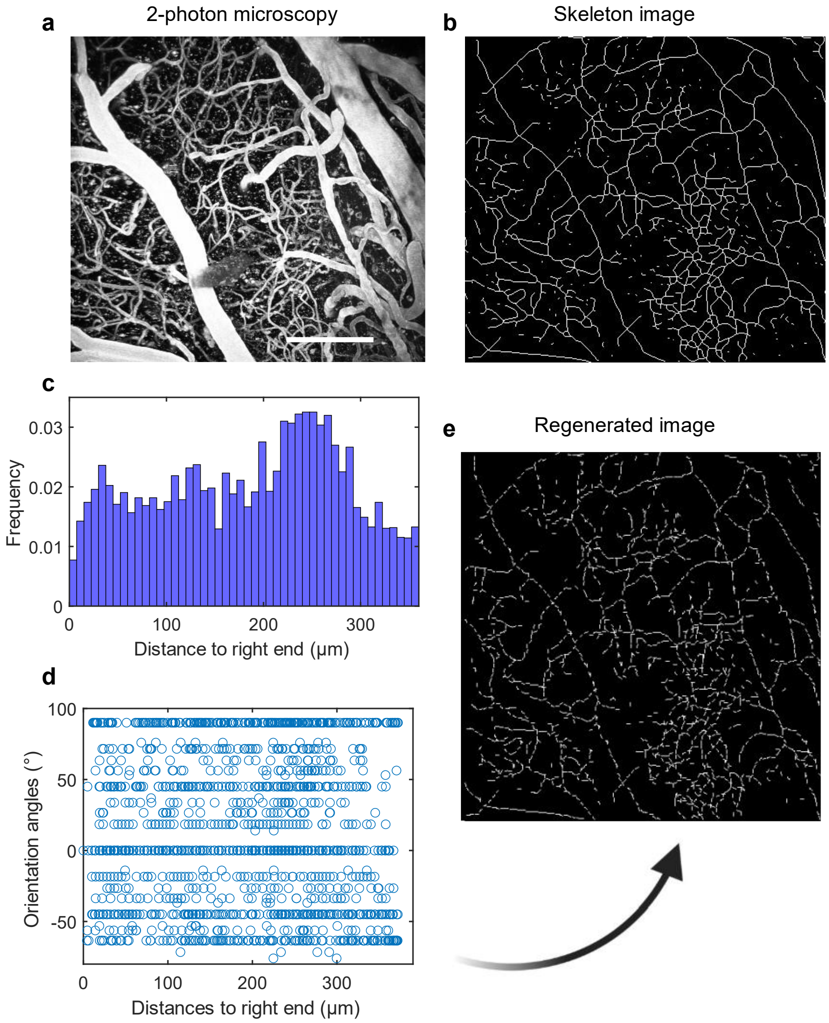

Supplementary Figure 12. **2P imaging of pial vasculature and extraction of vessel orientation**. **a.** Microscopy image of mouse vasculature. The field of view is 460 μm * 460 μm square shape. Scale bar, 100 μm. **b.** Post processed image after despeckle, binary transformation and skeleton transformation, via ImageJ software. **c.** Distance histogram, it represents the number of vessels at each specific distance from the right end of the image. **d.** Combined angle and distance distribution of vessels, it represents the orientation of blood vessels at each specific distance from the transducer. **e.** Image of the vasculature map that has been generated with an original MATLAB

code, only using the information from the angles and distances that were extracted from the original image.

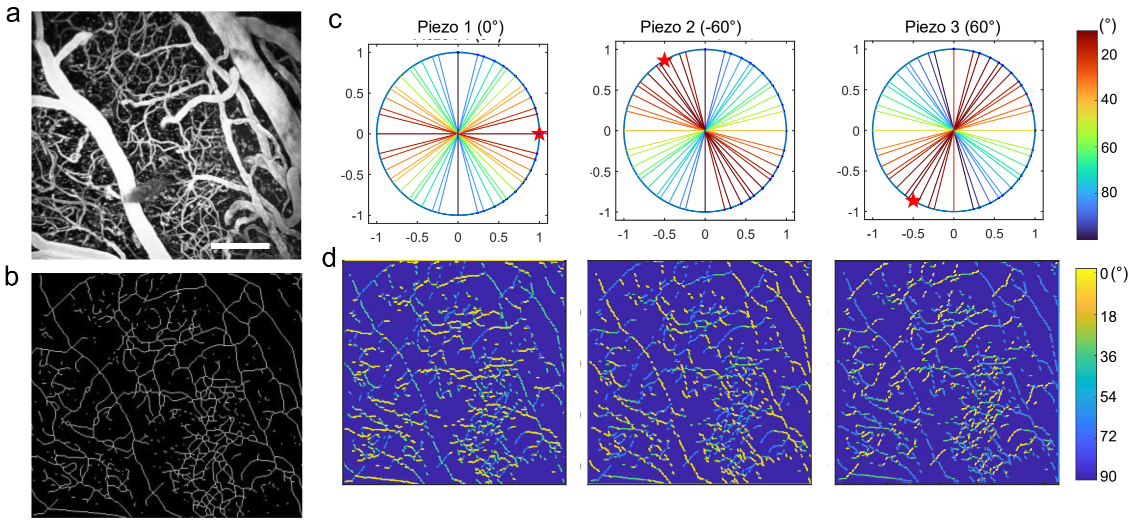

Supplementary Figure 13. **Prediction of swarm velocity and directionality from vasculature map images, example with three transducers. a.** Microscopy image of mouse brain vasculature. The field of view is 460 µm * 460 µm square shape. Scale bar is 100 µm. **b**. Post processed image after despeckle, binary transformation and skeleton transformation, via ImageJ software. **c.** Swarm velocity map derived for each transducer in the system. We have chosen polar coordinates to illustrate the orientation of blood vessels in space. Red stars mark the position of our transducers, to visualize their relative position with respect to the vessel's orientations. An angle of 0° indicates that the line connecting the image center to the transducer is parallel to the line following the vessel. While angle 90° indicates that they are perpendicular. Based on the relative orientation between transducer and vessel, we have used a colormap where we find in red the vessels where the swarms will move faster (higher influence of the acoustic wave), and in blue the vessels with lower velocities. **d.** Image of the vasculature map that has been generated with an original MATLAB code using the information from the angles and distances that we extracted from the original image. Specifically, here we have focused on the relative orientation between these vessels and the transducer. Brighter colors mean vessels in which higher swarm velocities are found, while darker (bluer) colors represent vessels with low or non microswarm movement.

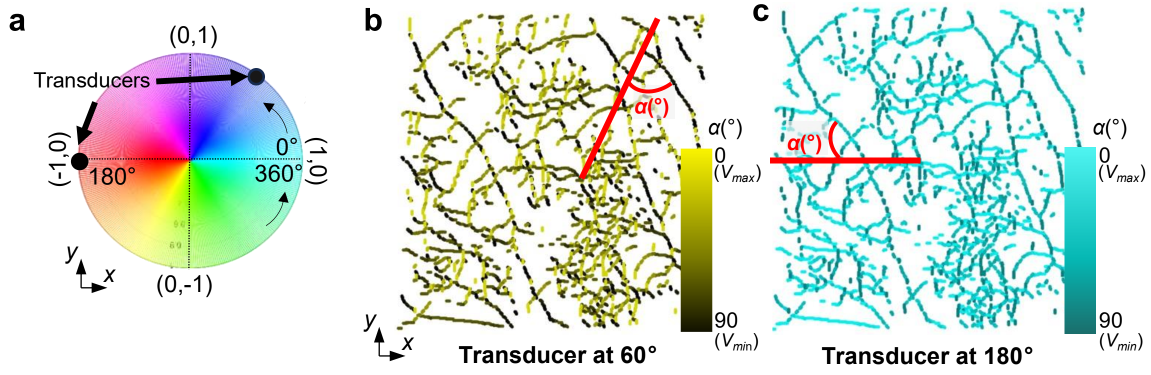

Supplementary Figure 14. **Prediction of microrobot movement direction based on transducer position. a.** Schematic of the colormap used to describe microrobot movement directionality. The direction of microrobots has been described by vector units which have their (0,0) at the center of the acquired image. Each vector unit has been assigned to a color, leading to a polar coordinate colormap system. The black dots on the graph indicate the locations of the transducers employed for images b and c. When referring to the position of the transducer angle, the line connecting (0,0) to (1,0) signifies an angle of 0°. Our measurements of angles follow a counterclockwise direction. Thus, the two black dots on the graph correspond to angles of 60° and 180°. **b, c.** Reconstruction of the vasculature network where the color of the vessels represents the direction where microrobots would move upon transducer activation. Different intensities (light or darker color) have been used to represent those vessels where movement is expected to occur more (V_{max}) or less (V_{min}) efficiently. Transducers at 60° and 180° have been used respectively. In the red line we have marked how the angle, α is calculated.

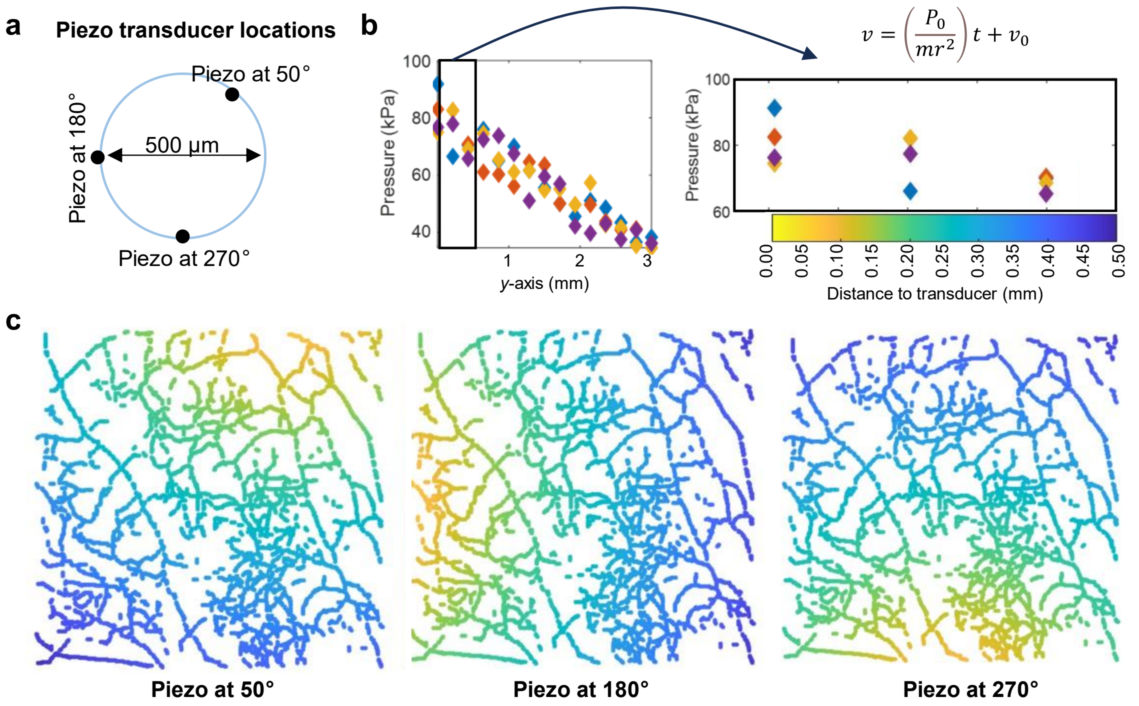

Supplementary Figure 15. **Prediction on microrobot velocity changes due to their distance from the transducer. a.** Schematic showing the position of transducers. Each black dot represents one transducer. To encompass the entire field of view, the maximum distance of the visualization window (measured diagonally) has been selected as the diameter of the circle within which the transducers are positioned, 500 µm. **b**. In this plot we present the decay of acoustic pressure versus distance, specifically focusing on the range of 500 µm. Each data point is a single measurement of pressure, and different colors indicate independent experiments. This range represents the pressure values within our field of view. The relationship between acoustic pressure and microbubble velocities, *v*, is depicted in an equation above the plot. Additionally, we provide a colormap representation below the plot, indicating the color assigned inside the software to each distance value. This colormap specifically corresponds to the color coding used in 'c'. **c.** Reconstruction of the vasculature network for the activation of three different transducers. In each case, the color of the vessels represents their distance to the respective activated transducer. This color scheme allows for a visual representation of the varying distances between the transducers and the vasculature network. Yellow means closer distances while blue means larger distances.

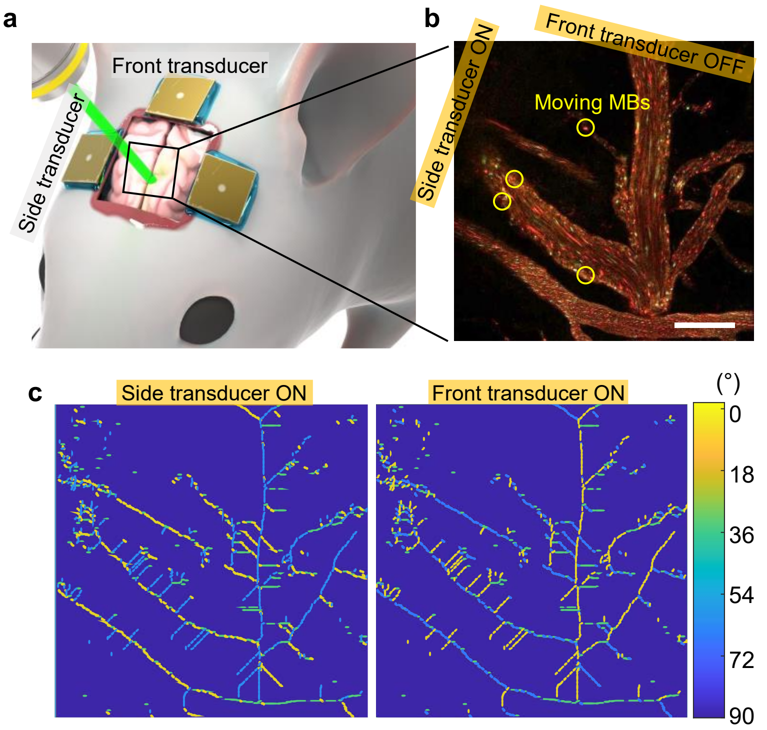

Supplementary Figure 16. **Prediction validation during in vivo experiments in pial brain vasculature. a.** Schematic of transducer positions on the mice. During this experiment we used two transducers that we labeled as front and side transducers. **b.** Image from the 2P microscope, the relative position of the transducers is shown. In yellow circles we marked the microbubbles that have formed swarms and that are moving due to the activation of the side transducer at 40 VPP and 490 kHz. Scale bar is 50 µm. **c.** Prediction images. These images have been generated with the developed software, and it represents the prediction of the most affected vessels upon activation of side transducer and front transducer respectively. Brighter colors mean vessels in which higher swarm velocities are found, while darker (bluer) colors represent vessels with low or no microswarm movement.

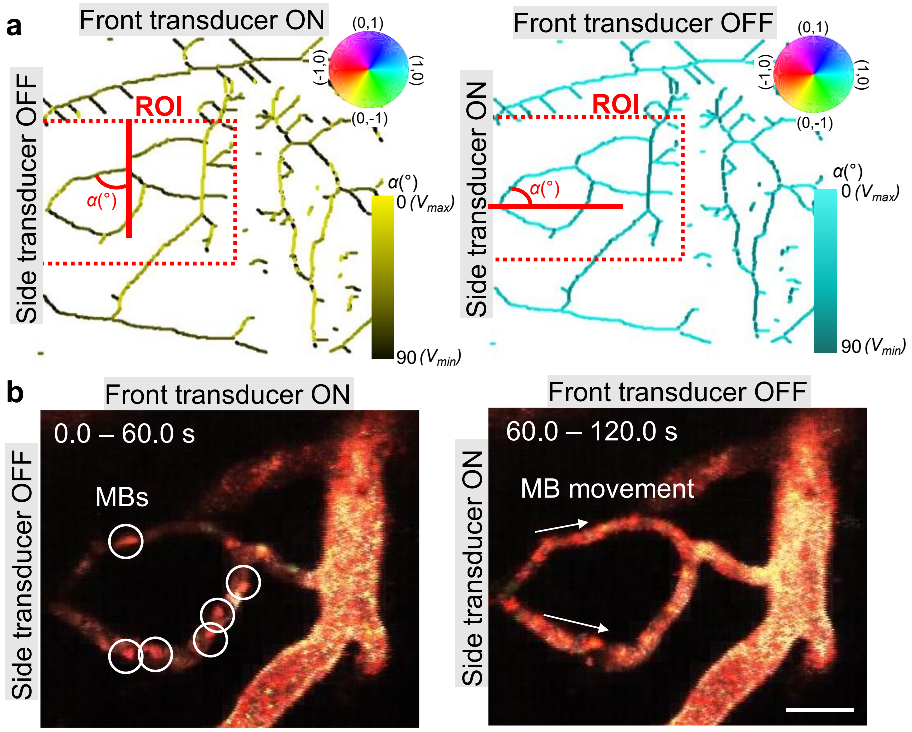

Supplementary Figure 17. **In vivo validation of microrobot trajectory predictions.** In this figure left images coincide with front transducer activation, while right images coincide with side transducer activation. See positions of front and side transducers in Supplementary Fig. 16. **a.** Reconstruction of vasculature lattice. The color assigned to each vessel indicates the trajectory that the microrobots will follow as they move within these vessels. To assist in interpreting the colormap, a reference is provided on the right side of the visualization (see also Supplementary Fig. 14). The color intensity (darker or brighter color) indicates those that will exhibit faster (v_{max}) or slower (v_{min}) microswarm navigation when activated by the transducers. This enhanced navigation is attributed to the relative orientation of these vessels with respect to the activated transducer, defined by the angle α. In the red line we have marked how the angle, α is calculated. **b.** 2P images from the mouse brain vasculature upon activation of front and side transducer respectively. During activation of front transducer, the microswarms are not present within vessels where navigation is efficient, so no movement is observed (see Fig. 4 for further explanations). During activation of side transducer, the microswarms show movement in the predicted direction. Validation results were reproduced for 5 independent experiments. Scale bar is 50 µm.

Manipulation of microrobots in vivo at far distances from the transducer.

We successfully validated the manipulation capabilities of microrobots at regions within the cranial window that are far from the activated transducer. To achieve this, we conducted imaging at the lower end of the cranial window (refer to the red square in Supplementary Fig. 18a) while activating Piezo transducer 2, positioned at the upper end of the cranial window (~2.7 mm distance between transducer and ROI 2). Through this experiment, we demonstrate that a single transducer can generate sufficient acoustic pressure to move microrobots with working distance up to 2.7 mm in any region of the window. The results revealed microbubbles forming swarms and navigating in the direction of wave propagation when the transducer was activated at 45 V_{PP} and 490 kHz.

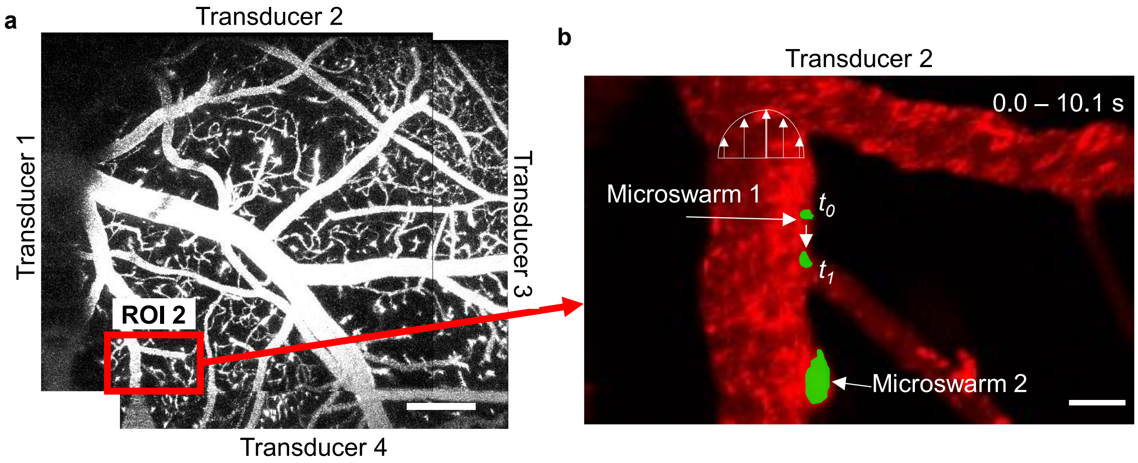

Supplementary Figure 18. **Validation of microrobot formation and navigation at distances far from the activated piezo transducer. a.** Microscope image of the cranial window vasculature network. We used 4 transducers for microbubble navigation inside this network and we show their location relative to the acquired image, see red squares. Scale bar is 200 µm. **b.** We show the formation of two different microbubble swarms labelled as microswarm 1 and 2. Microbubbles in microswarm 1 are manipulated down, by piezo transducer 2. Microbubbles in microswarm 2 self-assemble at the wall. No navigation was seen for this group of microbubbles. After image processing (see Methods), the microswarms have been colored in green while non-responding single microbubbles that flow downstream have been colored in red. This image results from a stack of 2 different frames taken between 0 and 10.1 seconds of video recording. This experiment was reproduced 3 times. Scale bar is 20 µm.

Cases of incipient vessel clogging

Microbubbles used for the experiments are much smaller than the vessel diameters, however, upon acoustic activation, the formation of swarms can result into sizes that come close to the diameter of the blood vessels, and thus clogging becomes an important factor to consider.

We experienced the appearance of incipient clogging events; these were identified by a sudden reduction in the flow velocity of a vessel and the presence of an accumulation of microbubbles. We characterized the number of times that we saw these events during in vivo experiments, compared to the total number of vessels analyzed, and we calculated a total of 3% of vessels that presented this situation. Importantly, after turning off the acoustics, the accumulation of microbubbles was cleared from the vessel, see Supplementary Fig. 19, and normal blood flow was recovered after some time.

We characterized the size of the vessels where these situations happened; we observed a dominance of capillaries with a size range between 1 and 10 μm in diameter, see Supplementary Fig. 19.

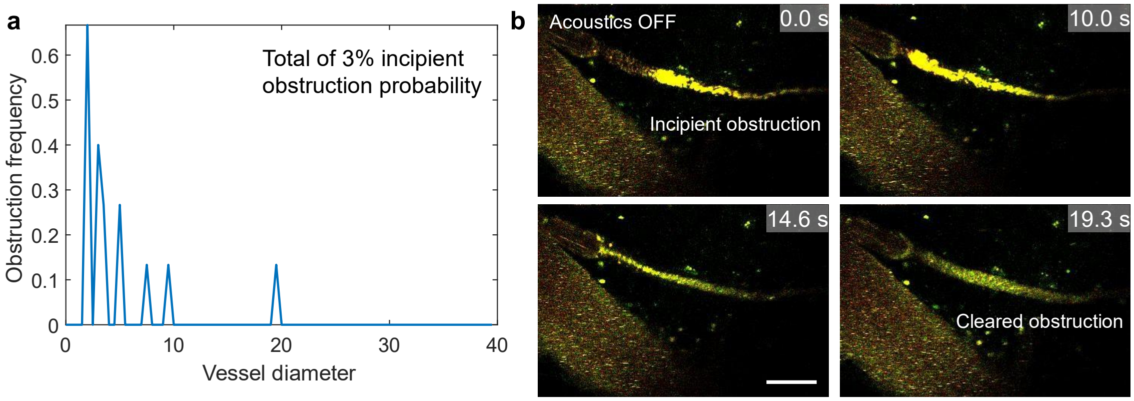

Supplementary Figure 19. **Cases of incipient vessel clogging. a**. Plot that shows the size distribution of those vessels that presented incipient vessel clogging. We can see the dominance of small vessels like capillaries and venules. **b.** Microscope image taken with the 2P microscope during a clogging event. Vessels shown are venules. Microbubbles are seen as bright yellow. After turning off the acoustics, microbubbles were washed away, and normal flow was recovered. This event was reproduced in 5 independent experiments. Scale bar, 50 μm.

Assessing clustering efficiency in veins, arteries and capillaries.

To assess the efficacy of microrobot control in vivo, we analyzed how successful was microrobot formation and navigation in blood vessels, and we studied how the vessel intrinsic characteristic (type, size and blood flow) affects the amount of microrobot control. For all in vivo experiments, acoustic signal was kept constant at 490 kHz and 44 V_{PP}.

First, we want to illustrate how higher blood flow values make more difficult microswarm formation. Higher blood flow means higher drag forces that compete with acoustic radiation forces, thus more difficult microbubble aggregations. The calculation of the drag force experienced by a spherical particle in a microscale flow can be performed using the Stokes drag formula, represented by $\mathrm{F\_drag} = 6\pi\mu a V$ (10). In this equation, **F_drag** denotes the drag force, *μ* represents the viscosity of the fluid, *a* stands for the radius of the particle, and **V** corresponds to the velocity of the fluid relative to the particle. Consequently, blood vessels with lower flow velocities generate lower drag forces acting upon the manipulated microbubbles. This relationship is illustrated in Fig. 5 of the main manuscript, where the diameter of the vessels was highly correlated to velocities.

Consequently, we have calculated the percentage of bubbles forming clusters with respect to the total number of bubbles that flow through each vessel in a period of time. In the section 'Fluorescent microbubble dosage and biocompatibility' within the Supplementary Information, we have explained how we compute the number of bubbles in each formed cluster. During the in vivo recordings, blood flow is fast and manual counting of the number of flowing bubbles is challenging. However, we know the concentration of bubbles in blood (based on total amount of bubbles injected and the total blood volume of the mouse), and we know the blood flows associated to each vessel diameter, so we can compute the number of bubbles that flow through a vessel during the recordings time. We have extracted the percentage of bubble clustering and we have plotted it against blood flow rates.

The experimental results show that the percentage of bubbles that form clusters during the recordings is lower in vessels with high blood flow, see Supplementary Fig. 20.

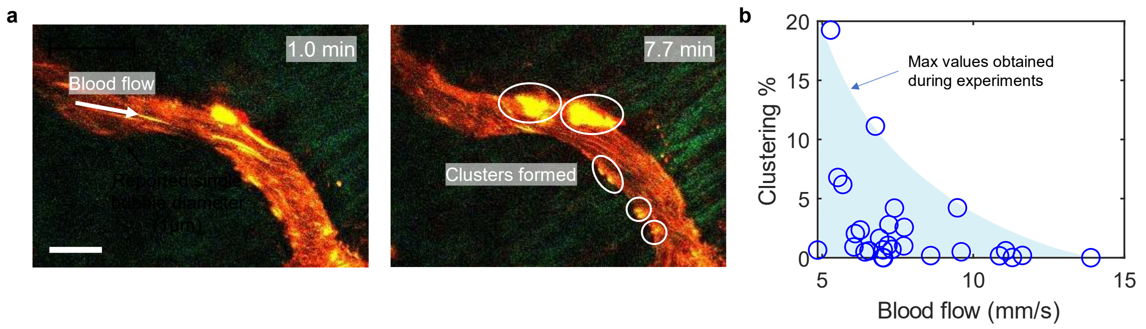

Supplementary Figure 20. **Microbubble formation versus blood flow**. **a.** 2P microscope images from the brain vasculature of the mouse showing microswarm formation over time. This behavior was reproduced in at least 10 independent recordings. The vessel shown is a venule. Microbubbles (bright yellow) formed at the vessel walls; and we marked with white circles some examples of swarms that we used for quantification of clustering %. Scale bar, 50 µm. **b.** Plot of the experimental results that show the percentage of clustering at each blood flow present inside blood vessels. Each circle measures the amount of clustering in one single vessel. Blue area illustrates the decay tendency of clustering % versus blood flow velocities.

We have additionally classified each vessel between venules, arterioles, veins and arteries, and we have plotted the frequency of microbubble clustering and navigation for each type of vessel. We have analyzed in total 240 formed clusters inside 25 different blood vessels, see Supplementary Fig. 21.

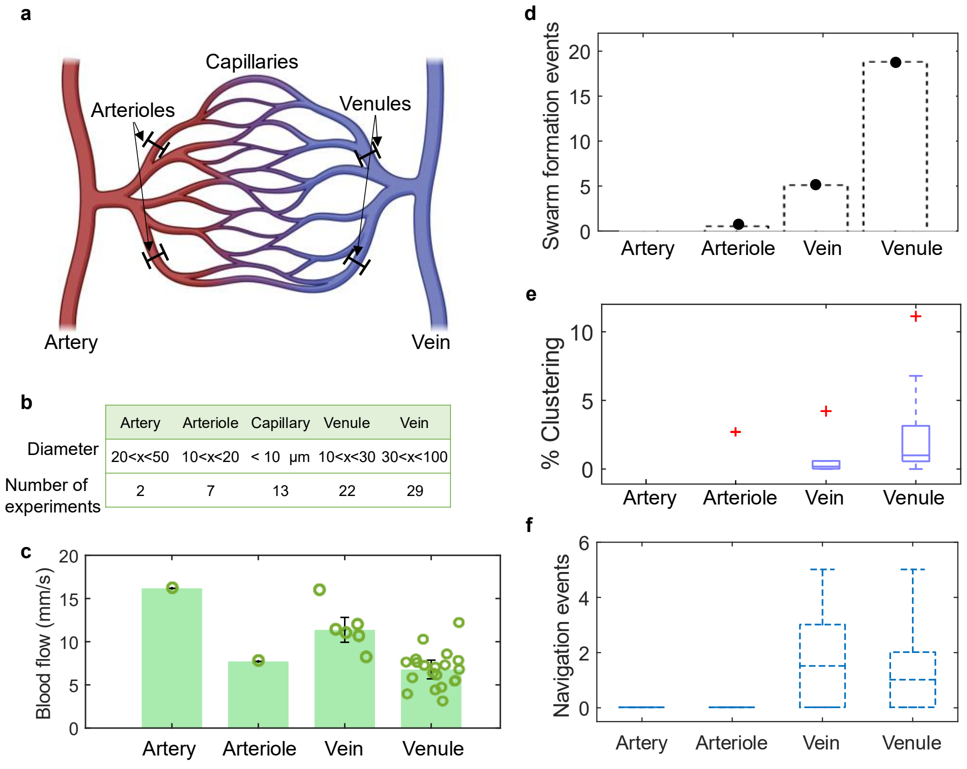

Supplementary Figure 21. **Study of swarm formation and navigation in arterioles, arteries, venules and veins.** The data from these plots has been extracted from a total of 240 formed clusters inside 25 different blood vessels **a.** Schematic representation of a capillary bed. Created with BioRender.com. **b.** Table shows the diameter range that we considered for each type of vessel, according to cited references and we count the amount of each vessel type that we imaged during experiments. A dominance of veins was imaged as they presented the higher chances for swarm formation and navigation. **c.** Blood flow measurements in different types of vessels. Each bar is the average of 1 to 18 independent measurements, which are shown overlaid in the graph as empty circles. **d.** Number of swarm formation events that were observed in each type of vessel. Each point represents a count during the analysis of 25 different vessels. **e.** Level of clustering in each type of vessel. We calculated the total percentage of flowing bubbles in each vessel that were responsive to acoustics and formed a swarm. n= 25 different vessels. The way we performed this calculation is detailed in the section ‘Fluorescent microbubble dosage and the level of acoustic response’. The middle line represents the median and the whiskers are the maximum and minimum values measured during experiments. The red cross indicates outliers. **f.** Number of swarm

navigation events observed in each different type of vessel. n= 25 different vessels. The middle line represents the median and the whiskers are the maximum and minimum values measured during experiments.

Analysis on the efficacy of microswarm navigation relative to microswarm formation

Microbubble swarm formation and navigation was not achieved with the same rate of efficacy. While swarms formed more easily, they tend to stay at the site of formation, ruled by their secondary Bjerknes interactions to the wall, and navigation was only achieved in certain cases. We have analyzed in each vessel the amount of swarm formation and swarm navigation, see Supplementary Fig. 22. It was observed that there is an increasing tendency between swarms forming and their navigation rate, the more swarms formed in a vessel, the more swarms presented navigation. However, if we pay attention to the values for swarm formation and navigation, swarm formation ranges between 0 and 25 swarms forming in a vessel, while navigation ranges between 0 and 5 swarms being navigated in a vessel. Note that navigation needs of higher acoustic energies to overcome the blood flow drag forces and to override the secondary Bjerknes adherence of the swarms to the wall, this contributes to the reduced amount of navigation of swarms compared to swarm formation.

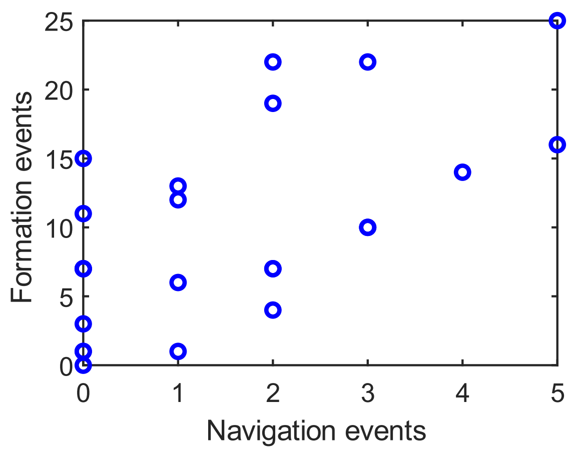

Supplementary Figure 22. **Efficacy of microbubble navigation relative to microbubble formation.** Plot showing the amount of microswarms that navigate inside a vessel compared to the

amount of swarms that form inside the same vessel. Each point represents a count from the analysis of 25 vessels and 240 microbubble clusters.

Measurement of blood flow inside blood vessels in vivo

To monitor blood flow, we used 2P microscopy. A line scan was conducted along the center of the imaged vessel, aligned with its direction. By examining this line scan, we were able to determine the flow velocities. This investigation was carried out across blood vessels of varying diameters to establish a correlation between vessel size and the corresponding blood flow. These findings were crucial for the subsequent analysis of microrobots.

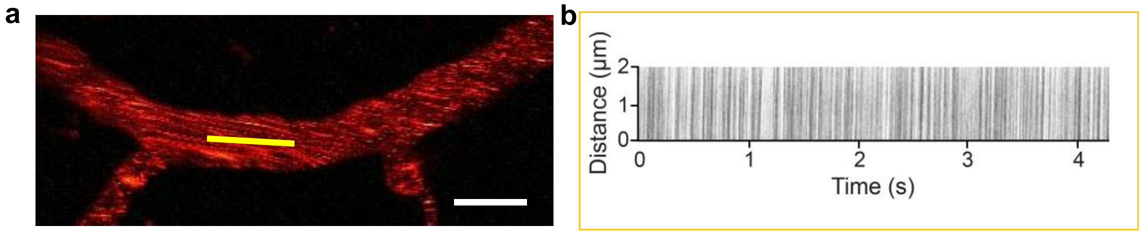

Supplementary Figure 23. **Blood flow measurements. a**. Image of a vessel imaged with 2P microscopy showing a line (yellow) drawn in the middle to perform a line scan and extract blood velocities. **b**. Results from the line scan, the plot shows distance versus time, each black line in the plot represents a flowing particle (i.e., red blood cells).

Biocompatibility studies

First, we have studied the effect of ultrasounds alone. A mouse C57BL/6 was coupled to two transducers, we applied 20V_{PP} for 3 recordings of 200 seconds each. Then 30V_{PP} for three recordings of 200 seconds each. And finally, 40 V_{PP} for 6 recordings of 200 seconds each. This is the same signal that was used for mouse during navigation experiments. During the recordings we didn't inject microbubbles, but we injected Hoechst 33342 Fluorescent Stain that stains cell nuclei and we proved that no leakage was observed during the whole experimental time. Fluorescent signal can be seen inside the vessels but not outside, proving endothelial integrity within the BBB.

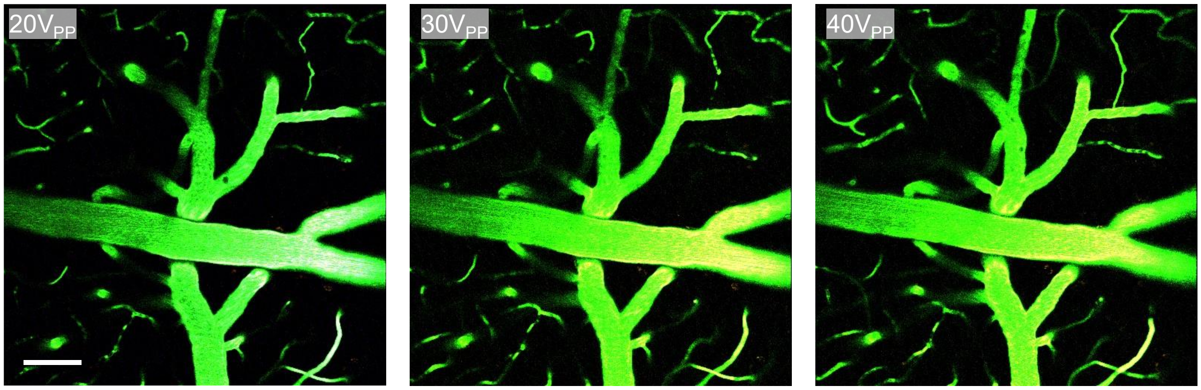

Supplementary Figure 24. **Ultrasound effect on BBB integrity**. Hoechst 33342 Fluorescent Stain was injected into the mouse, and increasing voltages were applied to two piezo electric transducers coupled to the mouse head. After 40 minutes of acoustic activation, no leakage or disruption of the BBB was observed, the dye remained inside the blood vessels. Scale bar, 50 μm.

Gas filled microbubbles that are surrounded by a lipid layer are often used as contrast agents in medicine, thus numerous studies have already proven their biocompatibility. We additionally assessed the biocompatibility of these bubbles for brain tissue. We have undergone biocompatibility studies to analyze the effect on brain tissue of oscillating microbubbles.

Histology results. We characterized neurons using NeuN staining, astrocytes via the marker GFAP, endothelial cells via the marker CD31 and cell viability using DAPI. Our results show that the acoustic activation of microbubbles does not trigger abnormal neuronal death. Endothelial cell lining keeps its integrity; however, astrocyte activation is present at the surface of the tissue, see Supplementary Fig. 25.

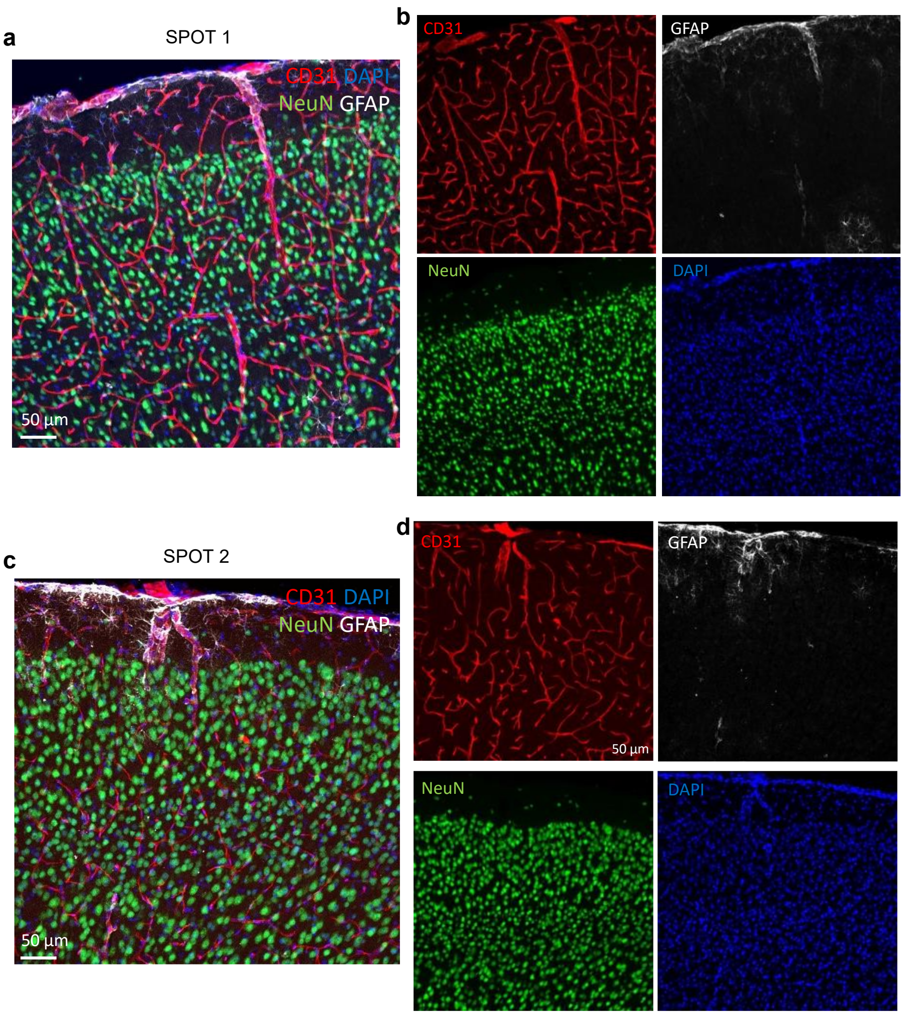

Supplementary Figure 25. **Histology immunostaining for biocompatibility analysis. a.** Merged image of a brain slice of the mouse after staining with CD31, DAPI, GFPA and NeuN. Scale bar, 50 µm. **b.** Individual images for each cellular maker, CD31, GFPA, NeuN and DAPI, from left to right, top down. The images show endothelial integrity, astrocyte superficial activation, neuronal survival and tissue integrity. **c.** Merged image of a brain slice of the mouse, at a second differentiated spot, after staining with CD31, DAPI, GFPA and NeuN. Scale bar, 50 µm. **d.** Individual images for each cellular maker, CD31, GFPA, NeuN and DAPI, from left to right, top down. The images show endothelial integrity, astrocyte superficial activation, neuronal survival and tissue integrity.

Measurement of temperature below ex vivo mouse skull.

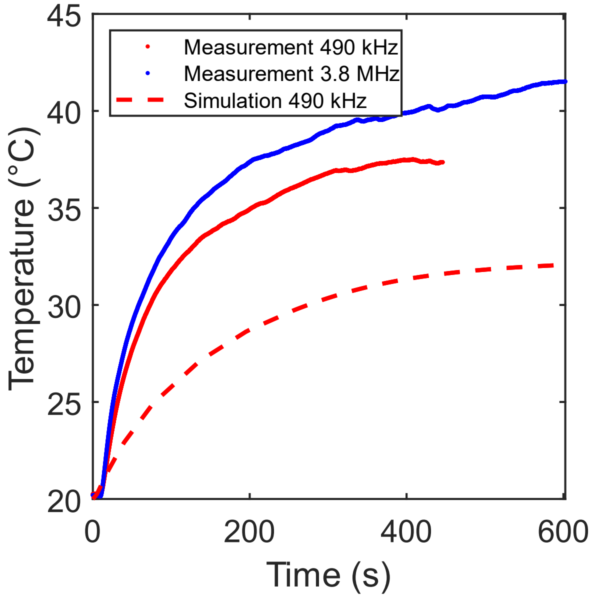

Supplementary Figure 26. **Temperature measurements.** Plot showing the increase of temperature caused inside water due to acoustic activation on top of an ex vivo skull. The piezo elements were excited with a square signal at the frequency of 490 kHz and with 20 VPP amplitude until the temperature reached equilibrium. We show measurements extracted from a type K thermocouple probe next to simulation results.

Supplementary References

1. Chugh, B. P., Lerch, J. P., Yu, L. X., Pienkowski, M., Harrison, R. V., Henkelman, R. M., & Sled, J. G. Measurement of cerebral blood volume in mouse brain regions using micro-computed tomography. *NeuroImage* **47**, 1312–1318 (2009).
2. Jenne, J. W., Preusser, T. & Günther, M. High-intensity focused ultrasound: Principles, therapy guidance, simulations and applications. *Z. Für Med. Phys.* **22**, 311–322 (2012).
3. Hynynen, K. & Clement, G. Clinical applications of focused ultrasound—The brain. *Int. J. Hyperthermia* **23**, 193–202 (2007).
4. Kim, Y., Rhim, H., Choi, M. J., Lim, H. K. & Choi, D. High-Intensity Focused Ultrasound Therapy: an Overview for Radiologists. *Korean J. Radiol.* **9**, 291–302 (2008).
5. Meng, Y., Hynynen, K. & Lipsman, N. Applications of focused ultrasound in the brain: from thermoablation to drug delivery. *Nat. Rev. Neurol.* **17**, 7–22 (2021).
6. Skeie, H. Electrical and Mechanical Loading of a Piezoelectric Surface Supporting Surface Waves. *J. Acoust. Soc. Am.* **48**, 1098–1109 (1970).
7. Ramakrishnan, N., Nemade, H. B. & Palathinkal, R. P. Resonant Frequency Characteristics of a SAW Device Attached to Resonating Micropillars. *Sensors* **12**, 3789–3797 (2012).
8. Patel, N. & Kirmi, O. Anatomy and Imaging of the Normal Meninges. *Semin. Ultrasound CT MRI* **30**, 559–564 (2009).
9. Strominger, N. L., Demarest, R. J. & Laemle, L. B. *Noback's Human Nervous System, Seventh Edition: Structure and Function* (Humana Press, 2012)
10. Embleton, T. F. W. Mean Force on a Sphere in a Spherical Sound Field. I. (Theoretical). *J. Acoust. Soc. Am.* **26**, 40–45 (2005).
11. Crocker, M. J. *Handbook of Acoustics*. (John Wiley & Sons, 1998).
12. Fonseca, A. D. C., Kohler, T. & Ahmed, D. Ultrasound-Controlled Swarmbots Under Physiological Flow Conditions. *Adv. Mater. Interfaces* **9**, 2200877 (2022)..
