# Supplementary material for: Ultrasound trapping and navigation of microrobots in the mouse brain vasculature
Source: Nat Commun. 2023 Sep 21;14:5889. doi: 10.1038/s41467-023-41557-3 (PMC10514062; doi:10.1038/s41467-023-41557-3)
Supplement: Supplementary file 3 — Description of Additional Supplementary Files [file 41467_2023_41557_MOESM3_ESM.pdf]

Description Of Additional Supplementary Files

Supplementary Movie 1 (separate file). Microswarm navigation to the wall and growth
(75.8 MB)
Within this movie we have two separated videos, they correspond to the results shown in Fig. 5 A,B. In both videos we can see microbubbles aggregating into bigger swarms at the vessel wall of a mouse brain. In both vessels there is a prominent blood downstream flow; nevertheless microbubbles are unaffected by it, and they aggregate and stay at the wall. We applied acoustic excitation at 490 kHz and 35 V peak-to-peak. The video was captured at 0.74 frames per second (fps) and played at 10 fps.

Supplementary Movie 2 (separate file). Microswarm upstream navigation inside in-vivo brain vasculature
(29.9 MB)
The video corresponds to Fig. 6B. The video shows the upstream navigation of a microswarm along the vessel wall of a mouse brain. We applied acoustic excitation at 490 kHz and 44 V peak-to-peak. The video was captured at 1.5 fps and played at 10 fps.

Supplementary Movie 3 (separate file). Microswarm upstream navigation and simultaneous growth
(45.2 MB)
The video corresponds to Fig. 6C. The video shows the navigation of a microswarm upstream along a vessel wall. On the way, the microswarm attracts more microbubbles and grows. The final microswarm is not only upstream, but also bigger than before. We applied acoustic excitation at 490 kHz and 35 V peak-to-peak. The video was captured at 0.74 fps and played at 10fps.

Supplementary Movie 4 (separate file). Microswarm navigation in 3D branched vessels
(40.7 MB)
The video corresponds to Fig. 7C. This video shows navigation of a swarm in a branched vessel trajectory. An inset on the left shows the vasculature within the cranial window and the relative position of the transducers. Four transducers were used for microbubble navigation through complex trajectories. On the right, we see the video of the navigation event. First, transducer 2 is activated, moving the swarm downwards, against the flow. Subsequently, transducer 2 is turned OFF and transducer 1 is turned ON, moving the microswarm into a smaller vessel to the right. The swarm moves to the right along the second vessel.

Supplementary Software
The MATLAB code generated in this study has been deposited in the Zenodo database, under the DOI: 10.5281/zenodo.8279585. License: Creative Commons Attribution 4.0 International.
Name of the file:
Prediction of microbubble-based microrobot navigation using acoustics inside mouse vasculature
This repository accompanies the manuscript 'Ultrasound trapping and navigation of microrobots in the mouse brain vasculature'. This repository contains scripts that perform 2D reconstructions of skeletonized vasculature networks. In our study we used acoustic transducers to manipulate microrobots inside the brain vasculature lattice. This software, given a chosen number of transducers, predicts microrobot formation in space, within the vasculature network. It also predicts microrobot trajectory and velocity inside blood vessels.
