# Supplementary figures and images for: Ultrasound trapping and navigation of microrobots in the mouse brain vasculature
Source: Nat Commun. 2023 Sep 21;14:5889. doi: 10.1038/s41467-023-41557-3 (PMC10514062; doi:10.1038/s41467-023-41557-3)

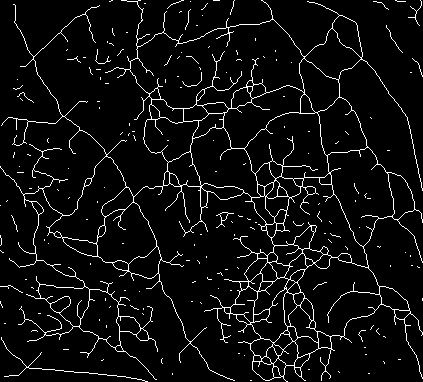

Supplement: Supplementary file 8 — Supplementary Software [file 41467_2023_41557_MOESM8_ESM.zip › software/software/Image reconstruction and spatial orientation analysis/veins1.tif]

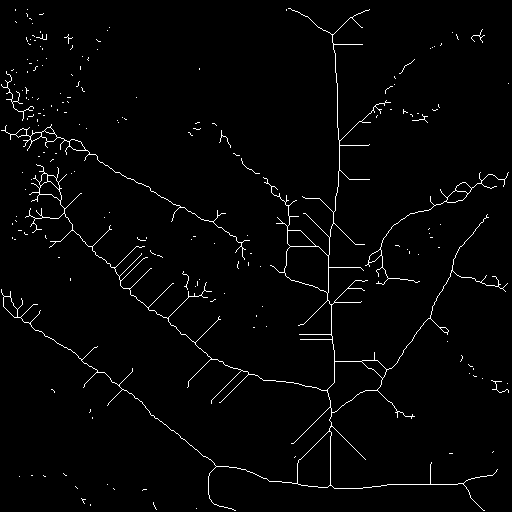

Supplement: Supplementary file 8 — Supplementary Software [file 41467_2023_41557_MOESM8_ESM.zip › software/software/Image reconstruction and spatial orientation analysis/veins2.tif]

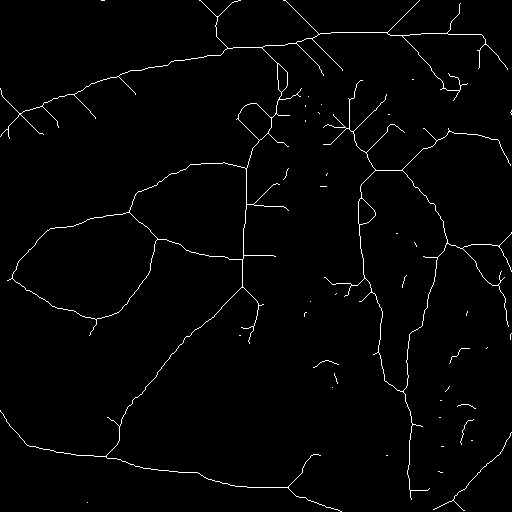

Supplement: Supplementary file 8 — Supplementary Software [file 41467_2023_41557_MOESM8_ESM.zip › software/software/Image reconstruction and spatial orientation analysis/veins3.tif]
