# Supplementary material for: Ultrasound trapping and navigation of microrobots in the mouse brain vasculature
Source: Nat Commun. 2023 Sep 21;14:5889. doi: 10.1038/s41467-023-41557-3 (PMC10514062; doi:10.1038/s41467-023-41557-3)
Supplement: Supplementary file 9 — Reporting Summary [file 41467_2023_41557_MOESM9_ESM.pdf]

nature portfolio

Corresponding author(s): Daniel Ahmed and Mohamad El-Amki

Last updated by author(s): 24/08/2023

# Reporting Summary

Nature Portfolio wishes to improve the reproducibility of the work that we publish. This form provides structure for consistency and transparency in reporting. For further information on Nature Portfolio policies, see our Editorial Policies and the Editorial Policy Checklist.

## Statistics

For all statistical analyses, confirm that the following items are present in the figure legend, table legend, main text, or Methods section.

| n/a | Confirmed | |
|---|---|---|
| ☐ | ☒ | The exact sample size ($n$) for each experimental group/condition, given as a discrete number and unit of measurement |
| ☐ | ☒ | A statement on whether measurements were taken from distinct samples or whether the same sample was measured repeatedly |
| ☒ | ☐ | The statistical test(s) used AND whether they are one- or two-sided<br>*Only common tests should be described solely by name; describe more complex techniques in the Methods section.* |
| ☒ | ☐ | A description of all covariates tested |
| ☐ | ☒ | A description of any assumptions or corrections, such as tests of normality and adjustment for multiple comparisons |
| ☐ | ☒ | A full description of the statistical parameters including central tendency (e.g. means) or other basic estimates (e.g. regression coefficient) AND variation (e.g. standard deviation) or associated estimates of uncertainty (e.g. confidence intervals) |
| ☒ | ☐ | For null hypothesis testing, the test statistic (e.g. $F$, $t$, $r$) with confidence intervals, effect sizes, degrees of freedom and $P$ value noted<br>*Give P values as exact values whenever suitable.* |
| ☒ | ☐ | For Bayesian analysis, information on the choice of priors and Markov chain Monte Carlo settings |
| ☒ | ☐ | For hierarchical and complex designs, identification of the appropriate level for tests and full reporting of outcomes |
| ☒ | ☐ | Estimates of effect sizes (e.g. Cohen's $d$, Pearson's $r$), indicating how they were calculated |

*Our web collection on statistics for biologists contains articles on many of the points above.*

## Software and code

Policy information about availability of computer code

| | |
|---|---|
| Data collection | Microscope recording was done with PVCAM 3.9.10.5 software package and in vivo two photon image was captured using ScanImage Janelia Research Campus R3.8.1 (RRID:SCR_014307) |
| Data analysis | The analysis of experimental data was done with ImageJ-Fji Version 1.53t, Microsoft Excel 365, MATLAB R2020b, MATLAB R2020b code CHIPS and Comsol 6.0. The MATLAB code generated in this study has been deposited in the Zenodo database, under the DOI: 10.5281/ zenodo.8279585. License: Creative Commons Attribution 4.0 International. Name of the file: Prediction of microbubble-based microrobot navigation using acoustics inside mouse vasculature |

For manuscripts utilizing custom algorithms or software that are central to the research but not yet described in published literature, software must be made available to editors and reviewers. We strongly encourage code deposition in a community repository (e.g. GitHub). See the Nature Portfolio guidelines for submitting code & software for further information.

## Data

Policy information about availability of data

All manuscripts must include a data availability statement. This statement should provide the following information, where applicable:
- Accession codes, unique identifiers, or web links for publicly available datasets
- A description of any restrictions on data availability
- For clinical datasets or third party data, please ensure that the statement adheres to our policy

The Source data generated in this study are provided in the Supplementary Information files. Source data are provided within an excel filed named 'Source Data'. This file contains experimental measurements that support all the graphs displayed in this article.
The MATLAB code generated in this study has been deposited in the Zenodo database, under the DOI: 10.5281/zenodo.8279585. License: Creative Commons Attribution 4.0 International. Name of the file: Prediction of microbubble-based microrobot navigation using acoustics inside mouse vasculature. The repository contains three .m files, three readme files with instructions to run the code and raw data for demo running.

## Research involving human participants, their data, or biological material

Policy information about studies with human participants or human data. See also policy information about sex, gender (identity/presentation), and sexual orientation and race, ethnicity and racism.

| | |
|---|---|
| Reporting on sex and gender | n/a |
| Reporting on race, ethnicity, or other socially relevant groupings | n/a |
| Population characteristics | n/a |
| Recruitment | n/a |
| Ethics oversight | n/a |

Note that full information on the approval of the study protocol must also be provided in the manuscript.

# Field-specific reporting

Please select the one below that is the best fit for your research. If you are not sure, read the appropriate sections before making your selection.

☒ Life sciences ☐ Behavioural & social sciences ☐ Ecological, evolutionary & environmental sciences

For a reference copy of the document with all sections, see nature.com/documents/nr-reporting-summary-flat.pdf

# Life sciences study design

All studies must disclose on these points even when the disclosure is negative.

| | |
|---|---|
| Sample size | No statistical methods were used to predetermine sample size for experiments. Sample sizes were based on reproducibility results, where we consider 5 succesful attemps as minimum requirement for each experiment.The results have been extracted from experiments on a total of 15 mice. From all the recordings, the presented data includes 39 different blood vessels and 200 different microbubble based microrobots. The reproducibility and the tendencies observed along the results suggest the sample size as sufficient to draw conclusions. |
| Data exclusions | No data exclusions were done |
| Replication | To verify the reproducibility of experimental results, the same experiments with the same experimental conditions and the same measurement were done a minimum of five times, with similar results in all of them. An average of these measurements was presented and the standard deviation shows the variability observed during the repetition of each experiment. For sample sizes below 10, each single data point is shown in the plot, while for sample sizes above 10 we have used box plots and violin plots. |
| Randomization | Not relevant to the study. The results describe the feasibility to manipulate microrobots in mouse vasculature, no control groups or randomized sample groups were used. All the mice had the same characteristics and they were considered as a single and unique sample. |
| Blinding | Blinding was not relevant to the study. All the mice were considered as the same sample, no blinding was needed |

# Reporting for specific materials, systems and methods

We require information from authors about some types of materials, experimental systems and methods used in many studies. Here, indicate whether each material, system or method listed is relevant to your study. If you are not sure if a list item applies to your research, read the appropriate section before selecting a response.

## Materials & experimental systems

| n/a | Involved in the study |
|---|---|
| ☐ | ☒ Antibodies |
| ☒ | ☐ Eukaryotic cell lines |
| ☒ | ☐ Palaeontology and archaeology |
| ☐ | ☒ Animals and other organisms |
| ☒ | ☐ Clinical data |
| ☒ | ☐ Dual use research of concern |
| ☒ | ☐ Plants |

## Methods

| n/a | Involved in the study |
|---|---|
| ☒ | ☐ ChIP-seq |
| ☒ | ☐ Flow cytometry |
| ☒ | ☐ MRI-based neuroimaging |

# Antibodies

| | |
|---|---|
| Antibodies used | The following antibodies and dilutions were used: anti-CD31 (cat. # ab56299, Abcam, 1:100), anti-NeuN (cat. # AB177487, Abcam, 1:1500) and anti-GFAP (cat. # ab4674, Abcam, 1:2000). Tissue sections were incubated with DAPI (4',6-Diamidino-2-phenylindole dihydrochloride) solution (stock 10mg/ml, 1:10000, cat. #D9542, Sigma-Aldrich,). |
| Validation | Every antibody utilized in this investigation underwent validation by its respective manufacturers, with detailed information available on the product websites. |

# Animals and other research organisms

Policy information about studies involving animals; ARRIVE guidelines recommended for reporting animal research, and Sex and Gender in Research

| | |
|---|---|
| Laboratory animals | For all experiments, C57BL/6j mice (Charles Rivers, no. 028), three to five months old were used. Both male and female mice were used in the imaging experiment. The mice had free access to water and food and an inverted 12-hour light/dark. The temperature ranged 23-25°, and the humidity from 40% to 60%. |
| Wild animals | The study did not involve wild animals |
| Reporting on sex | Sex of the animal was not relevant for the study. The mouse vasculature is independent on sex, so both male and female mice were used in the imaging experiment. |
| Field-collected samples | The study did not involve samples collected from the field |
| Ethics oversight | All animal experiments were approved by the local veterinary authorities in Zurich and conformed to the guidelines of the Swiss Animal Protection Law, Veterinary Office, Canton of Zurich. |

Note that full information on the approval of the study protocol must also be provided in the manuscript.
